# Atomic Structure, Stability, Raman Modes, and Electronic Properties of Quantum-Confined One-Dimensional Lepidocrocite Titanate and Water: A First-Principles Study

**DOI:** 10.1021/acsomega.5c10764

**Published:** 2026-01-30

**Authors:** Yuanren Liu, David Bugallo, Michel W. Barsoum, Yong-Jie Hu

**Affiliations:** Department of Materials Science and Engineering, 6527 Drexel University, Philadelphia, Pennsylvania 19104, United States

## Abstract

Quantum-confined, one-dimensional, 1D, lepidocrocite (1DL) titania nanofilaments are a recently discovered polymorph of TiO_2_ that holds great promise for various applications, including photocatalysis, water purification, dye degradation, and energy storage. These exceptional functionalities originate from 1DL’s unique atomic structure and diverse self-assembling morphologies, which are still under active investigation. Current understanding focuses on the atomic structure along the 1DL [100] growth direction, indicating that it shares a backbone atomic structure typical of two-dimensional lepidocrocite, 2DL, titania but exhibits significantly greater length along [100]. What has remained elusive is what the minimal achievable width along the [001] direction and why the 1DLs, despite their very small dimensions, are exceptionally water stable. In this work, the atomic structure and thermodynamic and dynamic stability of 1DL unit cells with varying [001] widths are investigated under a pH-neutral aqueous environment using first-principles calculations and ab initio molecular dynamics simulations based on density functional theory. We attribute the remarkable water stability to terminations induced by water molecules at the ribbon edges that tend to form hydrogen bonds between them when the number of water terminations is 4 per unit cell. Consequently, the theoretically minimal stable width of 1DL is found to be as small as only one lattice constant (2 TiO_6_ octahedra) of 2DL along the [001] direction. Additionally, the effects of cross-sectional width variation on the bandgap and Raman peak shifts are systematically studied and compared with those of 2DL to reveal quantum confinement effects induced by dimensionality reduction.

## Graphical abstract

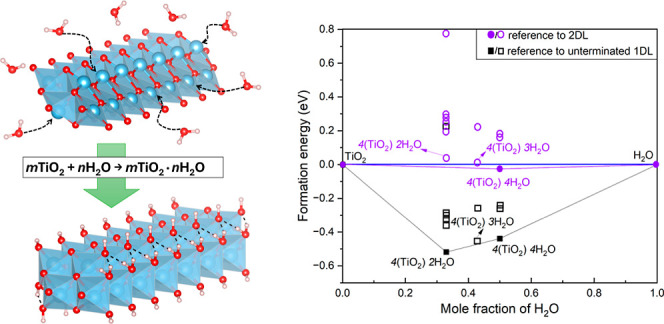

## 1 Introduction

Titanium dioxide (TiO_2_) is a widely used ceramic due to its combination of chemical stability, catalytic activity, low environmental impact, and natural abundance.^1,2^ Among the various polymorphs of TiO_2_, lepidocrocite stands out for its unique two-dimensional, 2D, layered crystal structure, where the TiO_6_ octahedra are all edge-sharing, which is distinct from conventional bulk polymorphs such as rutile and anatase.^3–5^ Henceforth, this 2D polymorph will be referred to as 2DL. The 2D lattice endows the material with high specific surface areas, SSAs, and excellent capacity for ion and molecule exchange, making it particularly well-suited for applications in catalysis,^6^ dye-sensitized solar cells,^7^ and batteries.^8–10^ These surface-related properties are further enhanced in nanostructured forms, where diverse morphologies such as nanosheets, nanowires,^11^ nanotubes,^12–14^ and nanoribbons^15^ have been synthesized. Nevertheless, these nanostructures are still all fundamentally composed of 2DL crystals. Further dimensional reduction is highly desirable, as it can dramatically enhance performance by further increasing the SSAs and introducing quantum confinement effects.

Recently, Badr et al. demonstrated a simple, one-pot, scalable method that realized for the first time the synthesis of titanate-based nanofilaments, NFs, that are truly one-dimensional (1D) starting with earth-abundant Ti-precursors.^16^ These NFs were initially observed to self-assemble into micrometer-sized flakes and were originally believed to possess an anatase-like atomic structure.^16^ However, subsequent characterizations—including X-ray diffraction (XRD), Raman spectroscopy, and scanning transmission electron microscopy (STEM)—confirmed that these NFs instead exhibited a backbone atomic structure similar to that of their 2DL counterparts.^17^ Notably, they displayed significantly greater lengths (20–30 nm) along the [100] direction relative to the other two lattice directions, thereby affirming their 1D nature. Based on this insight, we labeled these titanate NFs, 1DLs, which stand for 1D lepidocrocite titanate NFs. Note that the NFs are not titania in that their O/Ti > 2 and the excess negative charge is compensated for by cations.

Because of their reduced dimensions, the 1DL surface-related properties are significantly amplified. With an exceptionally high SSAs, of >1500 m^2^/g, 1DLs exhibit excellent capability of ion and molecule absorption, enabling promising applications in areas such as the removal of actinide cations from contaminated water,^18,19^ the formation of cation-stabilized hydrogels,^20^ and interface engineering for enhanced performance in perovskite solar cells and polymer composites.^21,22^ They are also applicable in Li–S and Li ion cells.^16,23^

The reduction in dimensionality also introduces a pronounced quantum confinement effect, resulting in bandgap energies, *E*_g_’s, as high as 4.5 eV in some cases^24^—significantly higher than all other previous TiO_2_ polymorphs. Intriguingly, and pertinent to this work, the *E*_g_ of filtered films could be increased by simply decreasing the colloidal suspensions, CSs, concentrations used to make them.^24^ Since the 1DL thicknesses and length were comparable, we hypothesized that as the concentration of CSs decreased, the widths of the ribbons in the filtered films decreased accordingly, resulting in a stronger quantum size effect.^24^ One of the impetuses for this work was trying to explain this novel effect.

Despite their high *E*_g_ values, 1DLs show great promise as efficient, sustainable, and low-cost photocatalytic materials. For instance, they exhibit a hydrogen production rate that is an order of magnitude higher than that of commercially used nanotitania, P25, powders.^46^ Furthermore, 1DLs demonstrate remarkable effectiveness in adsorption and subsequent photodegradation of some common cationic organic dyes.^26,27^ In the H_2_ case, we believe defect energy levels in the bandgap were involved.^25^ Remarkably, the 1DL NFs were stable for over 6 months in water/methanol mixtures, an observation that this work goes a long way in explaining. In the dye case, we showed that certain cationic dyes sensitized the 1DL NFs, allowing their degradation with the use of only visible light.^26,27^

These preliminary findings on the exceptional functionalities of 1DLs underscore the need for a deeper understanding of their atomic structure in order to establish clear structure–property relationships. To date, our previous studies have elucidated the atomic structure of 1DL NFs along the [100] and [010] directions. Specifically, along the [100] direction, 1DL retains a backbone atomic structure identical to that of 2DL, where the Ti atoms form zigzag chains with a translational periodicity of approximately 3.8 Å. Along the [010] direction, the backbone structure consists of just two edge-sharing TiO_6_ octahedra, yielding a thickness of ≈5.0 Å, i.e. one lepidocrocute sheet which is identical to that in 2DL. Through interactions with various interlayer cations, 1DLs can self-assemble into nanobundles with ordered stacking along the [010] direction. The stacking sequence depends on the cation species—exhibiting AAA stacking for Li^+^ and ABA stacking for Na^+^ and tetramethylammonium (TMA^+^) cations.^28^

Upon drying in different solvents, these nanobundles can further aggregate into a plethora of morphologies ranging from porous mesostructured particles, PMPs, to quasi-2D flakes.^49^ In contrast, the atomic structure and stacking mechanism along the [001] direction are not fully understood. Experimental efforts to characterize the [001] × [010] cross-section using high-resolution STEM face challenges due to the intrinsic 1D morphology, which complicates sample preparation. One of our previous studies suggested that the arrangement of Ti atoms at the cross-section of 1DL nanobundles follows a zigzag pattern, identical to that of 2DL.^28^ More recently, a charge density map, simulated from XRD patterns, indicated that the width of a single 1DL NF is as narrow as 5 × 7 Å^2^.^29^ Despite these insights, a comprehensive elucidation of the backbone structure and edge terminations along the [001] × [010] cross-section remains highly desired. In particular, identifying the minimal stable width along the [001] direction is essential, as it defines the fundamental building block for all 1DL-based nanostructures.

The patterns of recent small-angle X-ray scattering, SAXS, experiments of CSs, made by reacting TiB_2_ with TMAH at 80 °C for 5 d, could be well fit with parallelopipeds one lepidocrocite layer thick (≈0.5 nm), loosely self-assembled into 3–4 nm wide and longer than 30 nm ribbons.^30^ In a more recent paper, we reacted TiB_2_ with tetra propylammonium hydroxide, TPAH, for 4 d at 80 °C, and again fit the SAXS patterns with ribbons.^31^ In this case, the thickness remains the same, but the widths were ≈8–9 nm but still >30 nm long. Absence of distinct peaks or oscillations in *S*(*q*) implied that the ribbons remained predominantly isolated, with minimal aggregation. Lastly, it is crucial to note that in the CSs, along the *c*-direction, the NFs were self-assembled loosely; what was holding them together were not Ti–O–Ti bonds but, most probably, hydrogen bonds. Upon drying, the latter lose water and transform into wider ribbons or ultimately 2DL sheets.

Herein, first-principles calculations and ab initio molecular dynamics (AIMD) simulations based on density functional theory (DFT) were performed to investigate the thermodynamic and dynamic stability of 1DL unit cells with varying [001] widths. In particular, the effect of the aqueous environment on 1DL’s stability was implicitly modeled using the VASPsol package. It was found that the edge terminations induced by water, H_2_O, molecules on the [001] surfaces play an essential role in stabilizing the backbone structure of 1DL. The most stable termination configuration for 1DLs with varying widths was identified from a number of possibilities by thermodynamic convex hull analyses, followed by AIMD simulation for evaluating dynamic stability. Surprisingly, the theoretically minimal stable width of 1DL was found to be as small as only one lattice constant of the 2DL structure along the [001] direction. With the identified stable structures, the effects of cross-sectional 1DL width variations on the bandgap and Raman peak shifts were predicted and compared with those of 2DL to reveal quantum confinement effects induced by further dimensionality reduction.

## 2 Methods

### 2.1 Construction of 1DL Atomistic Models

As just noted, 2DL consists of edge-sharing TiO_6_ octahedra, maintaining periodicity along the [100] and [001] directions (Figure 1a). As evidenced by both STEM and XRD characterizations, 1DLs share the identical atomic structure with 2DL along the [100] and [010] directions.^17,28^ Along [100], the TiO_6_ octahedra form a zigzag, edge-sharing chain with a translational periodicity of approximately 3.8 Å. Along [010], both 1DL and 2DL are two TiO_6_ octahedra thick. Unlike 2DL, 1DL possesses a distinct 1D morphology. The growth predominantly occurs along the [100] direction, resulting in lengths (∼30 nm) significantly greater than dimensions along the other two crystallographic directions.^17^ The width of the 1DL structure along the [001] direction can vary from a few angstroms to several nanometers, depending on the synthesis and postprocessing conditions.^17,33^ Additionally, the STEM result further showed that the pattern of Ti atoms along [001] in 1DL is also identical to that in 2DL.^28^ Therefore, in this study, we construct a series of 1D unit cells based on the 2DL structure, aiming to elucidate the smallest stable 1DL atomic building block along [001]. As shown in Figure 1a, these 1D unit cells are derived by slicing an infinite 2DL lattice into atomic arrays with widths along the [001] direction varying from one to four times of the c-lattice parameter of the 2DL structure. Depending on their widths, hereafter these 1D unit cells will be referred to as 1C, 2C, 3C, and 4C structures whose cross sections are shown in Figure 1b and labeled as such. When inputting these unit cells for DFT calculations, periodicity is only preserved along the [100] direction. Vacuum regions are embedded along the other two directions to eliminate interactions with periodic images.

**Figure 1:**
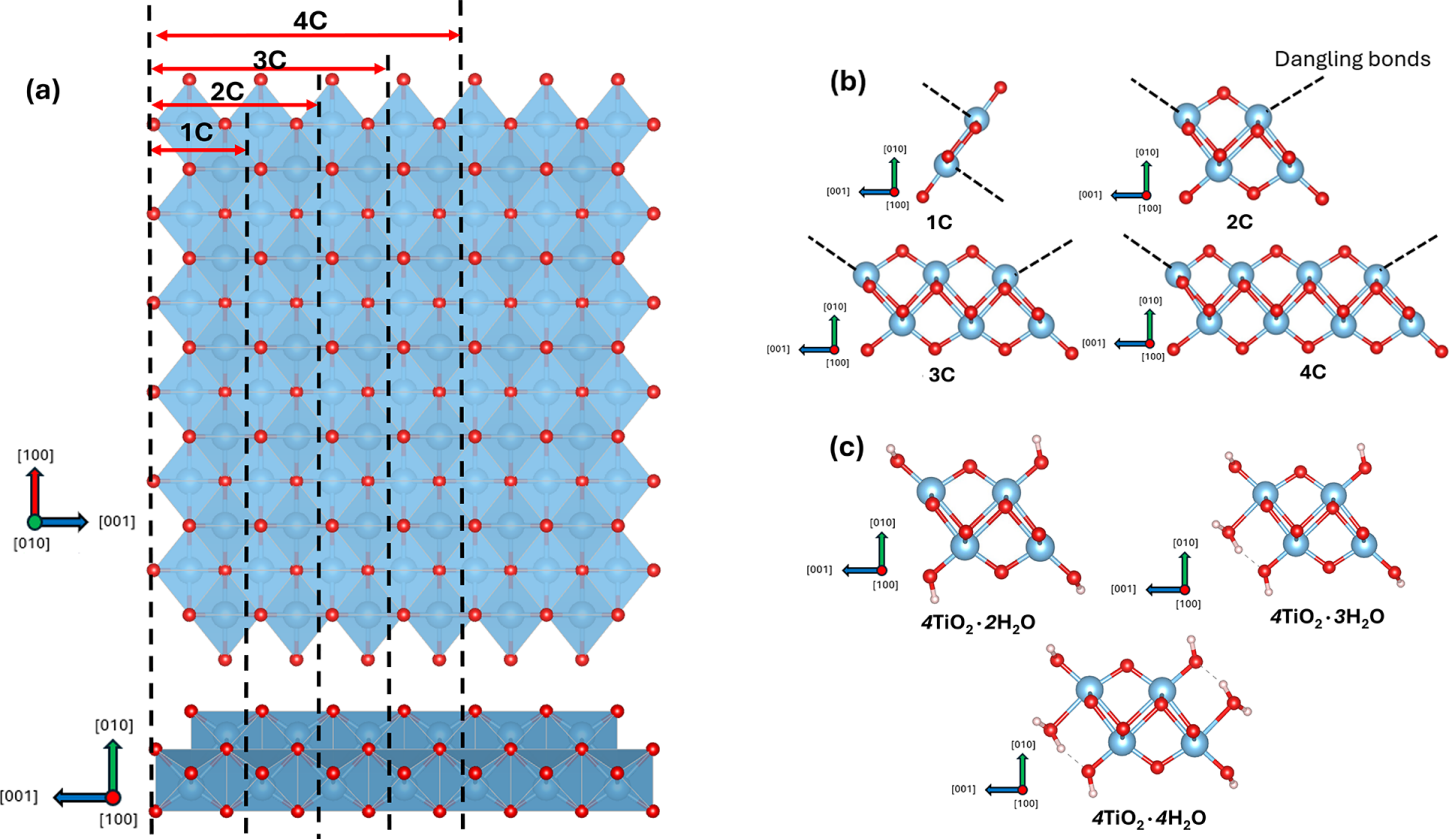
Origins of different 1DL widths: (a) slicing of an infinite 2DL sheet along [001]. Two projections are shown along [010] (top) and [100] (bottom), (b) 1D unit cells with different widths after cutting; edges of 1DL units possess dangling bonds depicted by dashed lines; (c) different 2C structures, with H^+^, OH^–^, or H_2_O balancing the dangling bonds. Blue spheres represent Ti, red, O, and white, H.

After slicing, and to preserve the TiO_2_ stoichiometry, dangling bonds—shown as dashed lines in Figure 1b—are inevitably introduced at the edges. Given that the synthesis and postprocessing conditions of 1DLs are to date aqueous, we considered healing these dangling bonds via interactions with H_2_O, as described by the following reaction

1 $$m{TiO}_{2}+nH_{2}O\to m{TiO}_{2}\cdot nH_{2}O$$

where *n* is the number of associated H_2_O molecules, and *m* increases with the [001] width of the 1DL structures. Specifically, the dangling bonds are stabilized by terminating the edge Ti and O atoms with H_2_O molecules or with pairs of –H^+^ and –OH^–^ groups to account for any dissociation of the H_2_O molecules. As such, charge neutrality is still preserved in the terminated 1D unit cell with the Ti and O ion valences remaining the classical +4 and −2 values, respectively. To identify the most energetically favorable termination configuration, several possible terminations, with *n* values ranging from 2 to 4, were systematically investigated. Figure 1c illustrates three examples of termination configurations (*n* = 2, 3, and 4, labeled as such on Figure 1c) for the 2C structure. A summary of all investigated unterminated and terminated configurations can be found in Tables S1–S8 in Supporting Information.

### 2.2 First-Principles Calculations and AIMD Simulations

The Vienna ab initio simulation package (VASP) was used for first-principles calculations,^34^ employing the projector-augmented wave method.^35^ Exchange–correlation effects were treated using the Generalized Gradient Approximation with the Perdew, Burke, and Ernzerhof functional.^36^ Wave functions were described using a plane wave basis with a cutoff energy of 520 eV. The Brillouin zone was sampled using the automatic meshing method implemented in VASP with a *R*_*k*_ value of 50 Å. This method adapts the grids of *k*-point mesh based on the length of reciprocal lattice vectors, resulting in a similar *k*-point density for the input structures with varying sizes. The threshold for structural relaxation was set to be 0.01 eV/Å. To model the effects of water solvent on the ground state energy and electronic structures of 1DL, the VASPsol package was employed. VASPsol is an implicit solvation DFT model that provides a computationally efficient method to calculate the electrostatics, cavitation, and dispersion effects of solvation on molecules and crystal surfaces.^37^ The input and relaxed atomic structure were visualized using the VESTA software.^38^

The VASP package was also employed for performing AIMD simulations using the exchange–correlation functionals and pseudopotentials used for the first-principles calculations.^34^ The *NVT* ensemble, where *N* is the number of particles, *V*, their volume, and *T*, their temperature, which were kept constant, was employed by using a Nosé–Hoover thermostat and a time step of 1 fs.^39,40^ The structural dynamics of the 1C, 2C, and 3C structures identified as thermodynamically stable were simulated for 4000 fs, during which the system temperature gradually increased from 0 to 300 K over the first 1000 fs and was maintained at 300 K for the remaining 3000 fs. For the 1C structure, an AIMD simulation was also performed at 368 K to evaluate its stability at a typical synthesis temperature. Snapshots of AIMD simulations were visualized using the OVITO software.^41^

For the stable 1DL unit structures, their electronic band structures were predicted using the hybrid exchange–correlation functional.^42^ The range–separation parameter was calibrated to reproduce the experimental bandgap energy *E*_g_ value of anatase TiO_2_ (3.2 eV). The energy convergence criterion for the self-consistent loop was set to be 10^–8^ eV. The Gaussian-smearing method, with a width of 0.05 eV, was used to process the integration in the first Brillouin zone. The VASPKIT package was employed to analyze the calculation results and generate plots.^43^

The theoretical Raman spectra were predicted for the stable 1DL unit structures based on first-principles phonon calculations. The finite displacement method was employed for the phonon calculations using 4 × 1 × 1 supercells, and the calculation results were analyzed using the Phonopy package.^44^ The Phonopy-Spectroscopy package was used to predict the Raman spectra based on phonon dispersions at the gamma point.^45^ For our phonon calculations, the VASPsol package was not employed for the sake of calculation simplicity.

## 3 Results and Discussion

### 3.1 Thermodynamic Stability

We found that the as-sliced, plain 1DL structures, i.e., those without termination on the (001) edges, are intrinsically unstable due to the presence of the dangling bonds, regardless of their width along [001]. After structural relaxation, the characteristic zigzag arrangement of edge-sharing TiO_6_ octahedra, typical of the 2DL lattice, either collapses entirely or becomes severely distorted. Detailed illustrations of these unstable and distorted atomic configurations are provided in Tables S1–S4.

As just noted, because 1DL synthesis typically occurs in aqueous environments, to compensate for the dangling bonds, we attached H_2_O molecules and/or their dissociated –H^+^ and –OH^–^ groups as terminations. According to eq 1—which can be interpreted as the product of a chemical reaction between bare TiO_2_ and H_2_O molecules—the chemistry of the terminated 1DL structures can be uniformly expressed as *m*TiO_2_·*n*H_2_O. Charge neutrality is preserved in these terminated structures, with Ti and O maintaining their classical valences of +4 and −2, respectively. In the *m*TiO_2_·*n*H_2_O formula, *m* characterizes the [001] width of the 1DL structure. Specifically, the 1C, 2C, 3C, and 4C structures correspond to *m* values of 2, 4, 6, and 8, respectively (see Figure 1b). As shown in Figure 1c, the number of H_2_O molecules, *n*, attached to the 1DL backbone, range from 2 to 4, representing surface terminations under either H_2_O-depleted or H_2_O-rich conditions, respectively. At each *m*TiO_2_·*n*H_2_O chemistry, multiple possible termination configurations (see Tables S5–S8) are evaluated to find the most stable ones.

To evaluate the thermodynamic stability of the 1DL structures with various termination configurations, convex hull analyses were conducted using formation energies, *E*_f_, derived from the chemical reaction described by eq 1. The *E*_f_ values were calculated by referencing the energies of solvent H_2_O molecules and bare TiO_2_ which is in the form of either unterminated 1DLs or an infinite 2DL sheet. Using the unterminated 1DL as a reference allows us to assess whether the interaction between 1DL and water molecules is energetically favorable. We additionally chose 2DL as a second reference state because it is the most stable edge-sharing TiO_6_ polymorph under ambient air conditions. Therefore, comparing the thermodynamic stability of 1DL relative to 2DL enables us to evaluate its phase stability in aqueous environments towards aggregation into the more stable bulk polymorph. For convex hull construction, the *E*_f_ of each termination configuration was plotted as a function of its H_2_O composition, expressed as *n*/(*m* + *n*). The resulting convex hulls for the 1C to 4C 1DL structures are presented in Figure 2a–d, respectively. Here, thermodynamically stable configurations are indicated by solid symbols; unstable/metastable ones are marked with open symbols.

**Figure 2:**
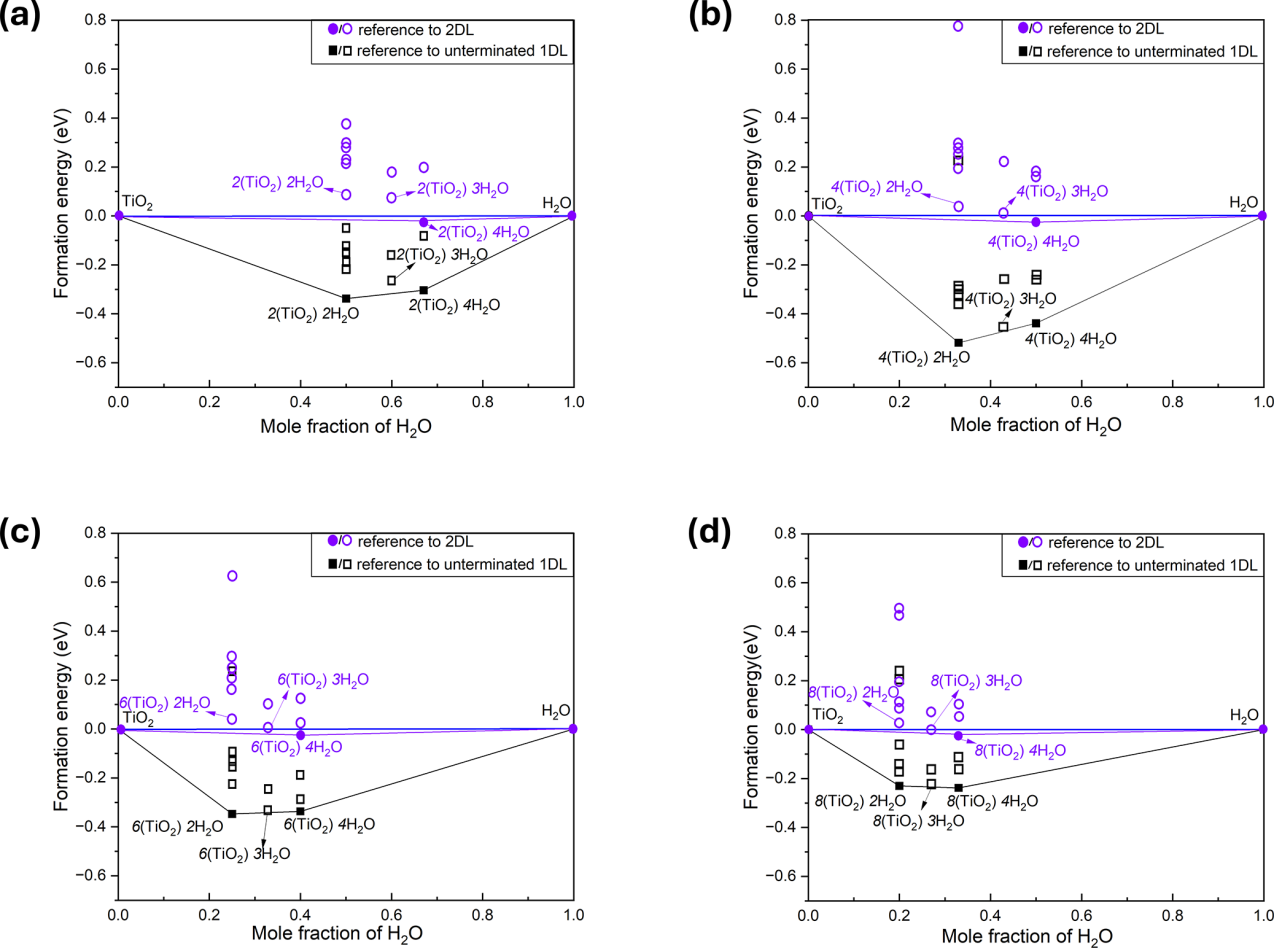
Convex hull analyses for, (a) 1C, (b) 2C, (c) 3C, and (d) 4C 1DL structures with various surface termination configurations. *E*_f_ (formation energy) was calculated using two sets of reference states: one based on the ground-state energy of the as-sliced, unterminated 1DLs (black squares), and the other based on the ground-state energy of 2DL (purple circles). Configurations on the convex hull are marked by solid symbols; the above-hull configurations are marked with open symbols. Chemical formulas for the thermodynamically stable or metastable configurations are labeled. Importantly, as shown by the solid purple circles, the 4H_2_O configuration is the *only* one more stable than 2DL, from the perspective of 0 K enthalpy of formation.

When referenced to the unterminated 1DLs, most of the terminated 1DL configurations exhibit negative *E*_f_ values indicating, not too surprisingly, that surface terminations are thermodynamically favorable. However, among all the configurations examined, two configurations consistently lay on the convex hull for the 1DLs with different [001] widths. One corresponded to an *n* = 2 configuration, in which the dangling bonds of the O and Ti atoms on the edges are passivated by two pairs of –H^+^ and –OH^–^ groups, corresponding to the dissociation of two H_2_O molecules. The other corresponds to an *n* = 4 configuration, where two additional H_2_O molecules are bonded to the edge Ti atoms of the *n* = 2 configuration. This restores the TiO_6_ octahedral coordination for all Ti atoms, including edge ones. Arguably, the most striking result in Figure 2 and Table 1, is that in all cases, and regardless of C, the 4H_2_O composition is the *only* one slightly more stable than 2DL (see solid purple circles in Figure 2) from the perspective of 0 K formation enthalpy. As argued below, this comes about because when 4H_2_O molecules terminate the NF edges, some of them are close enough to form hydrogen bonds.

**Table 1:**
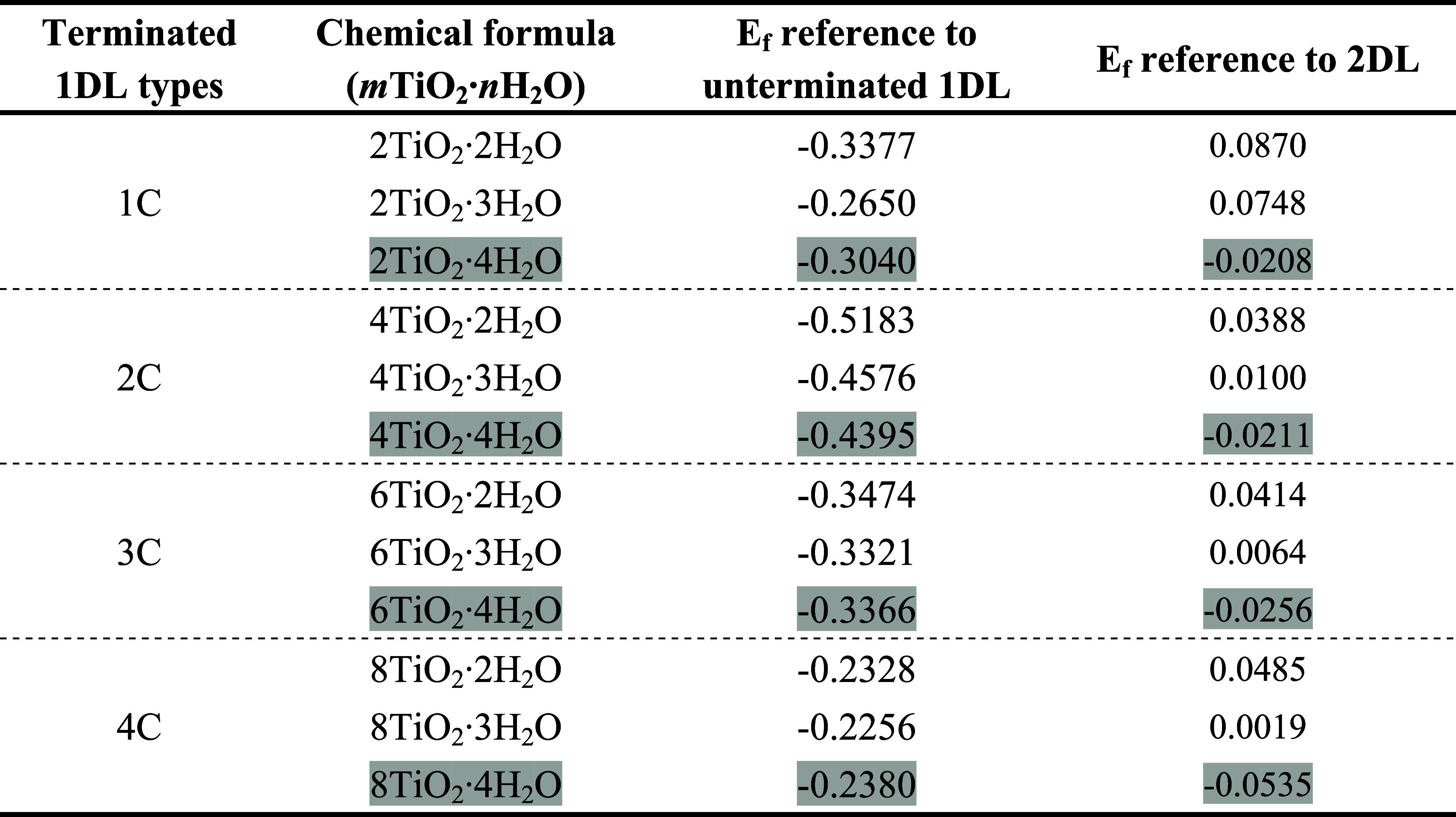
Chemical Formulae and Formation Energies of Thermodynamically Stable, or Slightly Metastable Terminated 1DL Structures Identified via Convex Hull Analyses^a^

a Energy unit is eV/molecule. Entries where *E*_f_ for 1DLs are lower than 2DL are highlighted in gray.

The *n* = 3 configuration, corresponding to an intermediate state between the *n* = 2 and 4 configurations, is found to lie very close to the convex hull (Figure 2), indicating its metastability. This also suggests that the stable *n* = 2 and 4 configurations can readily interconvert in response to variations in their aqueous surroundings, such as, for example, pH variations and mole ratio variations between 1DL and environmental H_2_O. In water, the only relevant configuration is n =4. The chemical formulas and *E*_f_ of these stable and metastable configurations for 1DLs with varying [001] widths are summarized in Table 1. To illustrate, the atomic structures of the *n* = 2, 3, and 4 configurations for the 2C 1DL structure are shown in Figure 3.

**Figure 3:**
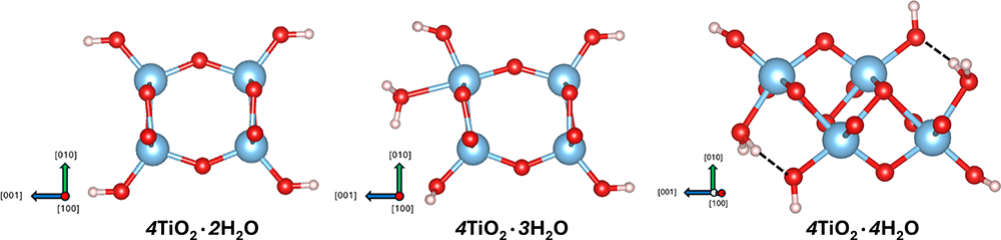
Cross-sectional atomic structures of thermodynamically stable and slightly metastable termination configurations for 2C 1DLs. Fine dashed lines in the far-right structure represent hydrogen bonds. The color scheme is the same as Figure 1.

As just noted when referenced to 2DL, only the *n* = 4 configuration (highlighted gray in Table 1) is found to be thermodynamically stable across 1DLs with varying [001] widths, as represented by the solid purple symbols in Figure 2. The *E*_f_ values are slightly negative and close to zero, implying that 1DLs possess comparable stabilities to their 2DL counterparts despite their 1D nature and significantly higher SSAs. Crucially, this is only true when water is introduced into the system. This important result suggests that in aqueous solutions, terminated 1DLs are quite stable with little, to no, thermodynamic driving force for aggregating into 2DL. This result is consistent with our experimental observations,^24,46^ and cannot be overstated because it indicates that 1DLs are most probably thermodynamically stable in water. As far as we are aware, 1DLs are the only highly water-stable quantum-confined 1D solids known.

To further assess the effect of 1DL widths on their stability, we consider the aggregation reaction

2 $$m_{1}{TiO}_{2}\cdot 4H_{2}O+m_{2}{TiO}_{2}\cdot 4H_{2}O\to \left(m_{1}+m_{2}\right){TiO}_{2}\cdot 4H_{2}O+4H_{2}O$$

This reaction represents the aggregation of two narrow 1DL NFs to form a wider one. The reaction is accompanied by the release of four H_2_O molecules through a typical polycondensation process.^47,48^ If the narrower 1DLs NFs were significantly less stable than their wider counterpart, this reaction would be expected to exhibit a large, negative *E*_f_. However, as shown in Table 2, the aggregation reactions among the 1C to 4C structures all yield slightly positive reaction energies. This result suggests that the 1DL stabilities, with varying widths, are comparable. Moreover, combined with the convex hull analysis relative to 2DL, these results suggest that 1DLs with different widths and terminated with 4H_2_O molecules (i.e., *m*TiO_2_·4H_2_O) likely coexist in aqueous environments and remain stable without spontaneously aggregating into much wider filaments or the 2DL crystal. Note this is only true in aqueous environments; the situation changes upon drying.

**Table 2:** Energies, in eV/formula Unit, of 1DL Nanofilaments Upon Their Aggregation into Wider Ones

aggregation reaction	reaction energy (eV/formula)
1C + 1C → 2C	0.081
2TiO_2_·4H_2_O + 2TiO_2_·4H_2_O → 4TiO_2_·4H_2_O + 4H_2_O	
1C + 2C → 3C	0.038
2TiO_2_·4H_2_O + 4TiO_2_·4H_2_O → 6TiO_2_·4H_2_O + 4H_2_O	
2C + 2C → 4C	0.054
4TiO_2_·4H_2_O + 4TiO_2_·4H_2_O → 8TiO_2_·4H_2_O + 4H_2_O	
1C + 3C → 4C	0.097
2TiO_2_·4H_2_O + 6TiO_2_·4H_2_O → 8TiO_2_·4H_2_O + 4H_2_O	

The atomic structures of the relaxed [010] × [001] cross sections for these stable, four H_2_O terminated 1DL structures are shown in Figure 4. Table 3 summarizes the cross-sectional dimensions, all of which fall within a few angstroms of each other. This supports their classification as truly 1D materials, especially considering that their practical lengths along the [100] direction typically range from 20 to 30 nm.^17,49^ Among these structures, the 2C 1DL (4TiO_2_·4H_2_O) exhibits a cross-sectional size consistent with values suggested by our previous experimental studies.^29^ Other configurations—including the 1C structure, which features an even narrower width—are also likely to coexist with the 2C one in aqueous colloidal suspensions, given their comparable thermodynamic stability in aqueous environments.

**Figure 4:**
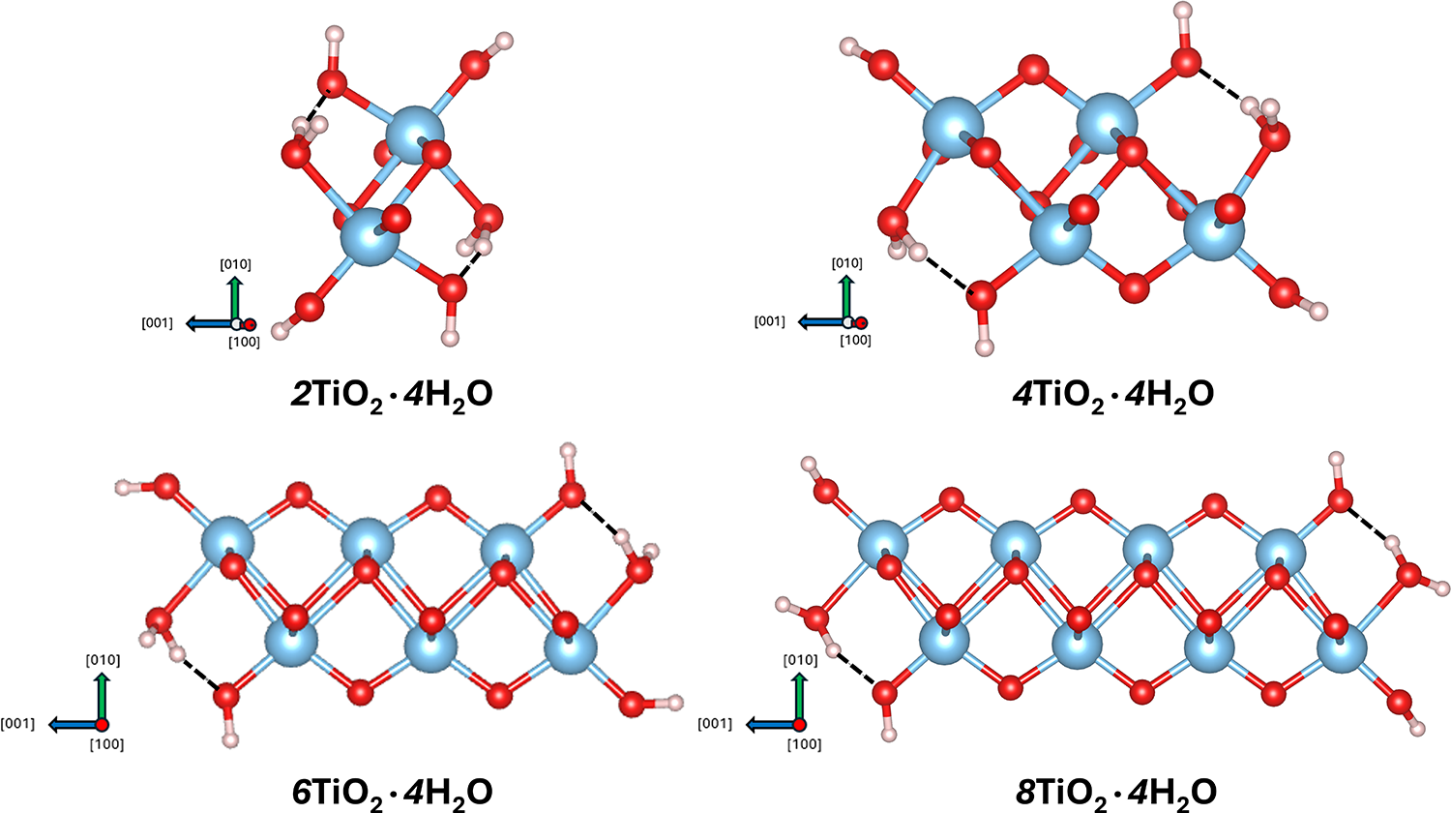
Atomic structures of [010] × [001] cross sections in stable, four H_2_O terminated configurations for 1DLs with different [001] widths. Dashed lines represent hydrogen bonds. The color scheme is the same as Figure 1.

**Table 3:** Structural Parameters of the Four H_2_O Terminated configurations for 1DLs with Different [001] Widths^a^

1DL types	chemical formula (*m*TiO_2_·*n*H_2_O)	width along [001] (Å)	thickness along [010] (Å)	O/Ti ratio	hydrogen bond length (Å)	hydrogen bond angle (degree)	interaction energy relative to *m*TiO_2_·3H_2_O (eV/formula)
1C	2TiO_2_·4H_2_O	4.9	6.0	4.00	1.66	164.3	–0.499
2C	4TiO_2_·4H_2_O	7.6	6.1	3.00	1.77	159.4	–0.239
3C	6TiO_2_·4H_2_O	11.6	6.1	2.67	1.78	162.5	–0.314
4C	8TiO_2_·4H_2_O	15.1	6.1	2.50	1.87	161.3	–0.264

a The interaction energy of these stable configurations relative to *m*TiO_2_·3H_2_O calculated based on eq 3 is also listed.

Figure 4 goes a long in explaining why the 4H_2_O termination is the most stable configuration. In this case, 2/3 of the edge oxygens are bonded to one H^+^ and the other 1/3 are bonded to two H^+^. In the equilibrium, or most stable configurations for the 4H_2_O chemistry, the H^+^ and OH^–^ terminations are close enough to each that they form hydrogen bonds, (depicted in all figures by short, dashed lines). The length and angles of hydrogen bonds in the 4H_2_O termination configurations with different [001] widths are summarized in Table 3. Those lengths and angles values fall in the typical range of solid-state O–H···O hydrogen bonds.^50,51,52^ Additionally, we also assessed the interaction energy of *m*TiO_2_·4H_2_O relative to *m*TiO_2_·3H_2_O via a reaction

3 $$m{TiO}_{2}\cdot 3H_{2}O+H_{2}O\to m{TiO}_{2}\cdot 4H_{2}O$$

As shown in Table 3, all reaction energies are negative, indicating that the *m*TiO_2_·4H_2_O configurations are thermodynamically more stable than *m*TiO_2_·3H_2_O. Noteworthily, the hydrogen bonds are not present in *m*TiO_2_·3H_2_O (Figure 3). Consequently, these negative reaction energies suggest that the formation of hydrogen bonds at termination can introduce a strong structural stabilizing effect. Additionally, the terminations in the *m*TiO_2_·4H_2_O configurations also restore the TiO_6_ octahedral coordination for all Ti atoms, including edge ones.

In these 4H_2_O configurations, singly bonded O anions running down the backbone alternate between those terminated with one and two protons. We note in passing that while in the 2DL structure the O anions are either bonded to 4 Ti^4+^ or 2 Ti^4+^ cations; in the 1DL structure, there are singly, doubly, triply and quadruply bonded O anions. The most important by far, from a termination and reactivity point of view, are the singly bonded O, that as far as we are aware are unique to the 1D structure.

To summarize this section, the emergence of hydrogen bonds is key to 1DL stability in water and explains quite elegantly why the four H_2_O configurations can be slightly more stable that even the infinite 2DL polymorph (highlighted entries in Table 1). This result cannot be overemphasized.

Before discussing the dynamic stability of these terminated, thermodynamically stable 1DL structures, it is worth noting that a fruitful, alternative way to think about their chemistry as follows. The chemistries of the 2H_2_O structures can be recast to read as

4 $$H_{4}{TiO}_{4}{\left({TiO}_{2}\right)}_{2n-1}$$

where *n* are integers, ranging from 1 for the 1C structure to ∞ for 2DL. Similarly, the 4H_2_O structure can be written as

5 $$H_{8}{TiO}_{6}{\left({TiO}_{2}\right)}_{2n-1}$$

The advantages of writing the chemistries as such are multifold. First, it emphasizes that in all cases the chemistry and structure of the surface terminations are identical; the only differences being the number of TiO_2_ slices that reside between the surfaces (Figure 1a). Second, the value of *n* essentially represents the [001] cross-section width of 1DL, where *n* = 1 corresponds to a width approximately one unit of the c-lattice parameter of 2DL, and so on, so forth for wider 1DL configurations. Third, it signifies that the O/Ti ratio is a function of *n*, (see last column in Table 3). This is quite important because it suggests that one can obtain a sense of the average widths of the 1DL ribbons by carrying out a careful X-ray photoelectron spectroscopic (XPS) study. Fourth, and as importantly, it confirms that since the O/Ti ratio is >2, the backbone must be negatively charged, with a charge that is compensated by 4 protons in eq 4 and 8 for eq 5. This negative charge is the reason that 1DL structures are readily ion exchangeable.^53^ Importantly, here the negative charge *per* Ti cation for eq 5, is −4 for *n* = 1 and −1.6 for *n* = 4, with the others in between. The corresponding values for eq 4 are half these values. Note that nondefective 2DL sheets, with *n* = ∞, are neutral. The reason they are charged has been ascribed to the presence of Ti vacancies.^54^

### 3.2 Dynamic Stability of Thermodynamically Stable 1DLs

The dynamic stability of the thermodynamically stable 1DL structures with 4H_2_O terminations was further evaluated at ambient temperature (300 K) using AIMD simulations. Figure 5a shows the potential energy and system temperature of 1C 1DL as a function of simulation time. Over a 3000 fs period, both potential energy, *E*_0_ and system temperatures only fluctuate slightly around constant values without sudden surges or irreversible changes, indicating that the 1C 1DL structure remains dynamically stable at 300 K. Figures 5b,c show snapshots of the atomic structure at the beginning (0 fs) and end (3000 fs) of the AIMD simulation, respectively. As shown, the TiO_2_ backbone remains structurally intact throughout the simulation, demonstrating its resilience to thermal vibrations at 300 K. On the other hand, throughout the simulation, some of the H_2_O terminations are observed to detach from the edge Ti atoms due to thermal perturbations, indicating the bonds between them are relatively weak. In contrast, no detachment of the –OH terminations was observed. Moreover, the 1C 1DL—despite its considerably narrow width of 4.9 Å—also exhibited dynamic stability even at the synthesis temperature (368.15 K) (Figure S1). Given that 1DL formation proceeds via a bottom-up process,^16,31^ it is not unreasonable to conclude that the 1C structure is likely to serve as a fundamental building block during its assembly.

**Figure 5:**
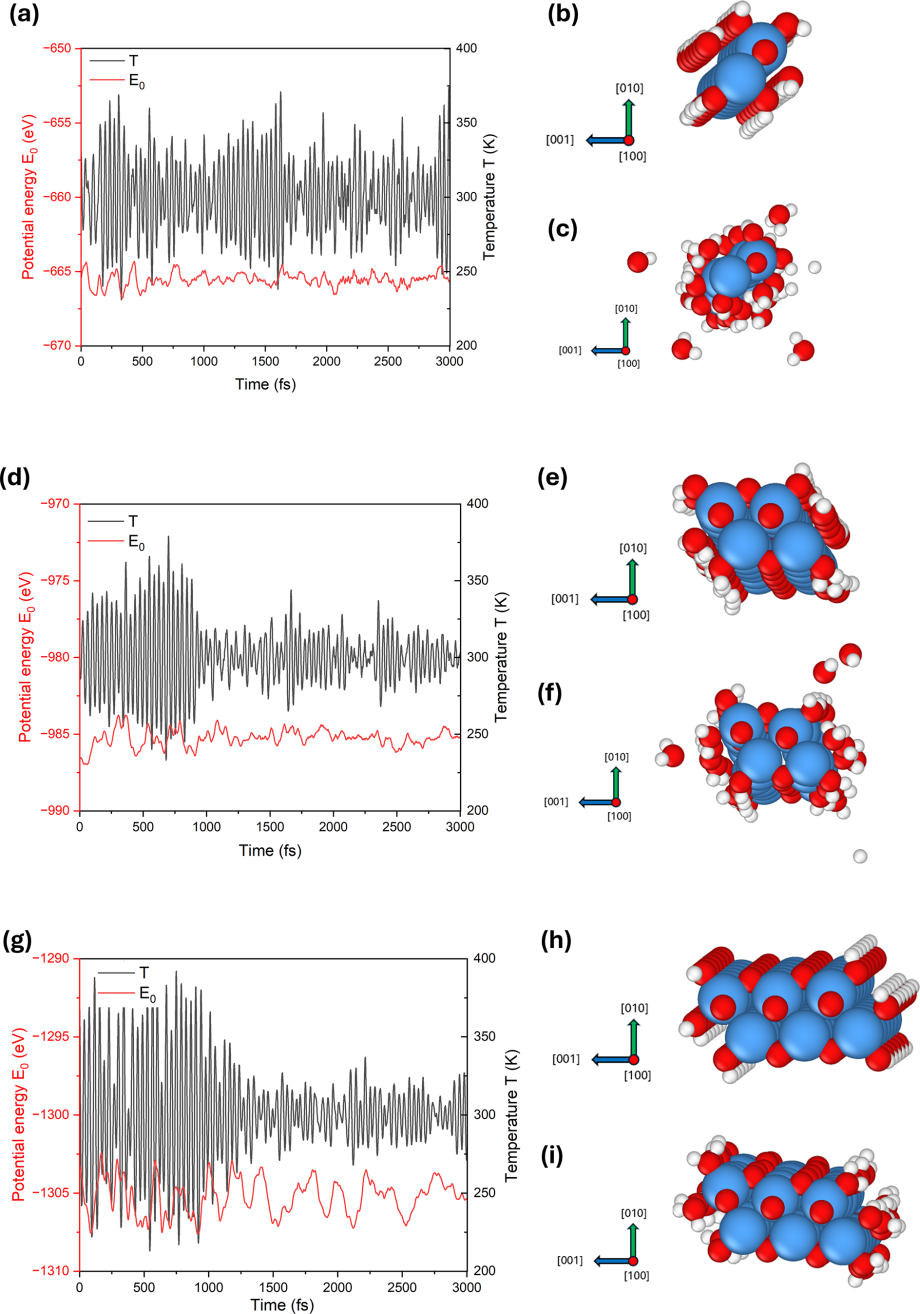
AIMD simulation of thermodynamically stable, 4H_2_O terminated 1DL structures. (a,d,g) Evolution of system potential energy and temperature as a function of simulation time for the 1C, 2C and 3C structures, respectively. (b,e,h) Snapshots of the 1C, 2C, and 3C atomic structures at the beginning of the simulation (0 fs), respectively. (c,f,i) Snapshots of the 1C, 2C, and 3C atomic structures at the end of the simulation (3000 fs), respectively.

The 2C and 3C structures are also found to be dynamically stable at 300 K, as shown in Figures 5d–i, respectively. In contrast to the 1C structure, only a few H_2_O molecules are detached from the 2C structure (Figure 5f), at the end of the simulation, and no detachment is observed for the 3C structure (Figure 5i). This suggests that an increase in the section width reinforces the termination bonds on the [001] edges, consequently enhancing structural stability.

The dynamic stability of a crystal structure is also commonly assessed by examining its phonon dispersions for the presence of negative frequency modes. In our phonon calculations, we observed small negative frequencies near the Γ-point. However, these negative frequencies do not indicate true dynamic instabilities but rather result from the vacuum regions introduced in the supercell to block self-image interactions, which in turn break the rotational sum rule of the force constants. Such spurious negative modes are frequently reported in studies of 2D materials and can be corrected using the Born–Huang condition and Huang invariances.^55^ In our previous work on 2D transition metal carbides (MXenes),^56,57^ we applied the hiPhive^58^ package for this correction, but found it difficult to extend to the 1D case. Therefore, in this study, we instead employed AIMD simulations to evaluate the dynamic stability of the 1DLs, a widely used alternative approach.^59,60^

### 3.3 Electronic Properties

The electronic properties of the stable 1C to 4C 1DL structures—including the electronic density of states (eDOS) and band structure dispersions—were predicted using hybrid exchange–correlation functionals. The total and projected eDOS, along with the band dispersion for the 1C structure, are shown in Figure 6a,b, respectively. The projected eDOS reveals significant overlap between the Ti d orbitals and O p orbitals, indicating strong Ti–O hybridization and suggesting that their bonding exhibits a partial covalent character in addition to its ionic nature, in agreement with previous DFT findings in other TiO_2_ polymorphs.^61^ Additionally, the overlap between the O p orbitals and H s orbital at lower energies corresponds to the covalent O–H bonds in the termination groups. As shown in Figure 6b, the 1C 1DL exhibits relatively flat valence maximum and conductive minimum bands, suggesting direct band gap behavior. The eDOS and electronic band structure of the 2C, 3C and 4C 1DL are shown in Figure S2, which exhibit similar characteristics to those of 1C 1DL.

**Figure 6:**
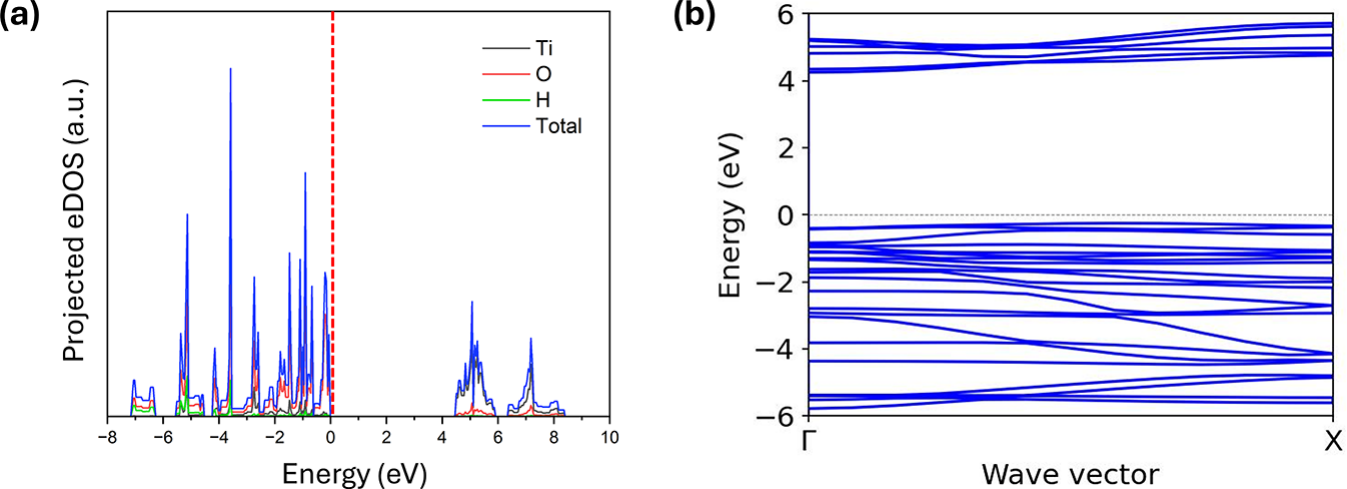
Electronic structures of 1C 1DL. (a) Total and projected eDOS and (b) electronic band dispersion. The red dashed line in (a) corresponds to the position of the fermi level.

To investigate the quantum confinement effect induced by dimensionality reduction, the band gap energies, *E*_g_, of the stable 1C to 4C 1DL structures were compared with those of 2DL. As shown in Figure 7, all 1DL structures exhibit larger *E*_g_ values than their 2D counterpart, highlighting the impact of the 2D-to-1D transition. Notably, *E*_g_ increases with decreasing 1DL width. In particular, the 1C structure exhibits an *E*_g_ as high as 4.5 eV, consistent with our recent experimental observation performed on filtered films made from extremely dilute aqueous 1DL colloids.^24^ Moreover, the UV–vis spectra of 1DL films filtered from colloid solutions were experimentally measured in our recent work.^24^ These UV–vis measurements revealed that *E*_g_ of the filtered films decreases from ∼4.5 eV to ∼3.5 eV as the concentration of 1DL in the colloid solution for filtering changes from 0.01 to 40 g/L. Combined with the trend observed in the theoretically predicted *E*_g_’s, it implies that at low colloid concentrations, the filtered 1DL filaments preferentially maintain a narrow cross-sectional width, whereas at higher concentrations they tend to aggregate into wider filaments.

**Figure 7:**
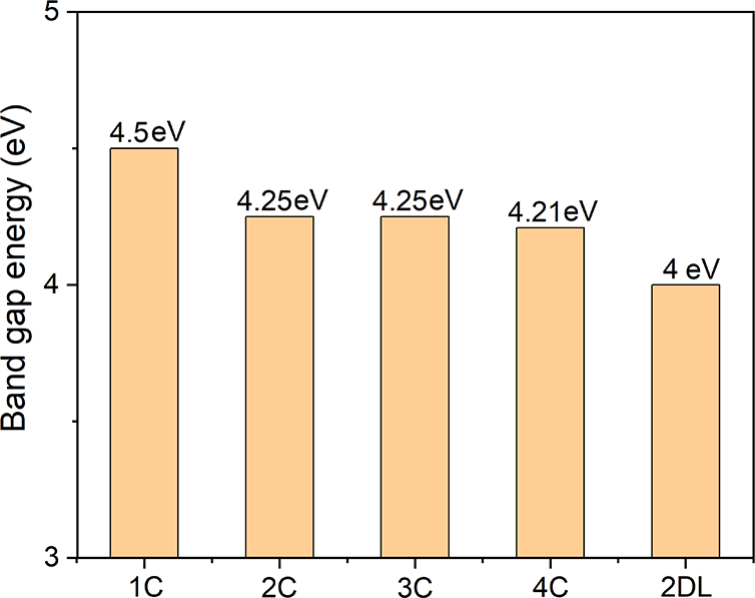
Band gap energies of stable 1C to 4C 1DL structures in comparison to that of 2DL.

While the predicted *E*_g_ values indicate that light absorption in the 1DL backbone structures occurs primarily in the UV region, the wide bandgap also confers a high oxidative potential. Moreover, it has been shown that the light absorption of 1DLs can be extended into the visible range by introducing intragap states.^25^ Combined with their superior aqueous stability, 1DLs therefore hold great promise for photocatalytic applications. For example, our recent work showed that 1DLs generated an order of magnitude higher H_2_ gas than their commercial TiO_2_ (P25) counterpart in water–methanol mixtures under UV irradiation.^46^ Furthermore, compared with their 2D and bulk counterparts, 1DLs offer a tunable bandgap that can be adjusted from approximately 3.5 to 4.5 eV simply by controlling colloid concentration during film filtration, providing a straightforward method to tailor electronic properties without resorting to chemical doping or complex synthesis routes.^24^

### 3.4 Theoretical Raman Spectrum

In nanomaterials, size effects, surface chemistry, and dimensional confinement can alter the vibrational properties of chemical bonds. These changes are typically reflected in the form of peak shifts and/or broadening in Raman spectra, which serve as sensitive fingerprints for identifying variations in bonding, composition, crystal structure, and dimensionality. Therefore, in this work, the theoretical Raman spectra are predicted for the stable 1DL structures to identify potential spectral fingerprints that could aid in their structural characterization. Figure 8 presents the predicted Raman spectra of the 2C and 3C 1DL structures, with 4H_2_O terminations, along with that of 2DL for comparison. Compared to the 2DL case, the 1DL Raman spectra generally have more peaks due to the breaking of symmetry and periodicity along the [001] direction. Among these peaks, two types of vibrational modes are identified: backbone vibrations (Ti–O framework vibrations) and termination vibrations (associated with edge terminations such as H_2_O molecules and –OH groups), which are marked by solid squares and open circles, respectively. It is noteworthy that the peaks associated with terminations may not appear or may get significantly broadened in the spectra measured experimentally, because, due to thermal perturbation, the actual configuration of edge terminations is likely to be less ordered and symmetric compared to the ground state configuration predicted by DFT calculations at 0 K.

**Figure 8:**
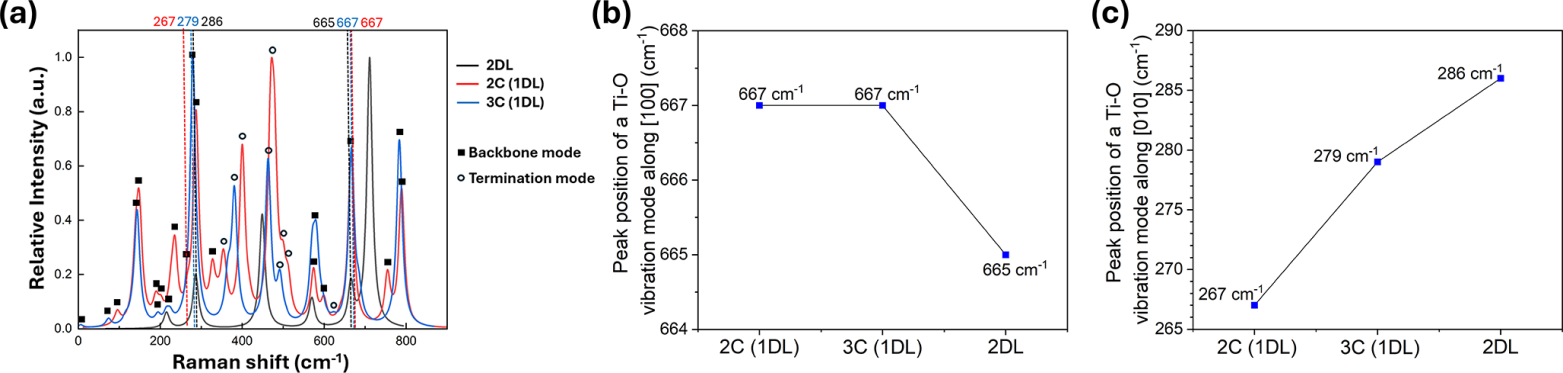
(a) Theoretical Raman spectra of 2C and 3C 1DLs 4H_2_O terminations, compared with those of 2DL. In the 1DL spectra, peaks arising from vibrations of the Ti–O backbone are marked with solid squares, while open circles indicate vibrational modes associated with surface termination groups. (b) Shift of Raman peaks corresponding to the stretching of Ti–O bonds along the [100] direction. (c) Shift of Raman peak corresponding to the stretching of Ti–O bonds along the [010] direction.

Our previous study has identified a Raman peak fingerprint that originates from the stretch of the Ti–O bonds in the backbone along the [100] direction.^24^ As shown in Figure 8b, this peak appears at 665 cm^–1^ in 2DL and shift to higher frequencies in 1DL. Additionally, the present work also finds a new Raman spectral fingerprint around 280 cm^–1^ for 1DLs. This fingerprint is associated with a Ti–O bond vibration along the [010] direction. As shown in Figures 8a,c, this peak shifts from 286 cm^–1^ in the 2DL to 279 cm^–1^ in the 3C 1DL structure, and further to 267 cm^–1^ in the 2C 1DL structure, showing a dependence on 1DL widths. This shift was also revealed in the experimentally measured Raman spectra in our previous work without an explicit discussion at that time.^24^ These theoretical findings are expected to offer valuable insights for future Raman-based structural characterization of 1DL-based nanomaterials.

## 4 Conclusions

In this work, we conducted a comprehensive first-principles investigation of the atomic structure, stability, and electronic and vibrational properties of 1DLs with varying widths along the [001] direction under a pH=7 aqueous environment. We demonstrated that as-sliced, unterminated 1DL structures are unstable due to dangling bonds on the [001] edges. 1DL stability can be significantly enhanced, however, through water-induced terminations, particularly those corresponding to a chemistry of H_8_TiO_6_(TiO_2_)_2*n*−1_, where n denotes the number of lattice units along the [001] direction. In this configuration, the dangling bonds at the (001) edges are passivated by two H_2_O molecules and two pairs of –H^+^ and –OH^–^ groups, which restores the octahedral coordination for all Ti atoms, including edge ones. Convex hull analyses indicated that these terminated 1DL structures are thermodynamically stable and comparable to their 2DL counterpart, under aqueous conditions, by the formation of hydrogen bonds between the now adjacent terminations. In this formalism, the O/Ti ratio is a function of n and varies from 4 to 2 as n increases to infintity. It follows that a measure of that ratio in, say, XPS can, in principle, yield valuable information about the average widths of the dried NFs.

The dynamic stability of these terminated 1DL structures was confirmed by AIMD simulations. While the theoretically minimal stable width of 1DL was found to be as small as only one lattice unit of 2DL along the [001] direction, aggregation analyses revealed that terminated 1DLs with different widths exhibit comparable thermodynamic stabilities, suggesting the potential coexistence of multiple NF widths in aqueous environments. Note that increasing the colloidal concentrations should increase the chances of wider NFs.

Theoretical Raman spectra were predicted to identify structural fingerprints of 1DLs. It was found that the 2D-to-1D structural transition resulted in characteristic shifts in the vibrational frequency of two Ti–O bonds in the backbone structure. First-principles electronic calculations based on hybrid functionals showed that 1DLs, with different widths, all exhibit larger *E*_g_s than those of titania and other TiO_2_ polymorphic phases. In particular, the 1C 1DL possesses a band gap as high as 4.5 eV, in good agreement with our experimental findings. These findings not only enhance our fundamental understanding of 1DLs but also provide valuable guidance for their experimental characterization and future application in photocatalysis, environmental remediation, and energy-related technologies.

## Supplementary Material



## Data Availability

The data of the atomic structures of the thermodynamically stable and metastable 1DLs are available to the public through Materials Commons, an open-access repository, via the doi link: http://doi.org/10.13011/m3-xnms-k920.

## Notes

The Supporting information contains tables and figures that summarize (1) atomic structures of unterminated 1DL structures; (2) terminated 1DL structures found to be thermodynamically unstable, along with their formation energies; (3) AIMD simulation results of the four H_2_O terminated 1C 1DL structure at synthesis temperature; (4) eDOS and band structure dispersion of 2C, 3C, 4C 1DL structures; and (5) details of the Raman mode in 2C and 3C 1DLs as well as 2DL (PDF)

## †

Decotek Automotive, Mullingar Rd, Collinstown, Co. Westmeath, N91 KHN1, Ireland

## References

[1] Kang X., Liu S., Dai Z., He Y., Song X., Tan Z. (2019). Titanium dioxide:
from engineering to applications. Catalysts.

[2] Haider A. J., Jameel Z. N., Al-Hussaini I. H. (2019). Review
on titanium dioxide applications. Energy Procedia.

[3] Zhang Y., Jiang Z., Huang J., Lim L. Y., Li W., Deng J., Gong D., Tang Y., Lai Y., Chen Z. (2015). Titanate and titania
nanostructured materials for environmental and
energy applications: a review. RSC Adv..

[4] Bavykin D. V., Friedrich J. M., Walsh F. C. (2006). Protonated titanates and TiO_2_ nanostructured
materials: synthesis, properties, and applications. Adv. Mater.:Compos. Carbon, Pap. Symp..

[5] Sasaki T., Watanabe M., Michiue Y., Komatsu Y., Izumi F., Takenouchi S. (1995). Preparation and acid-base properties
of a protonated
titanate with the lepidocrocite-like layer structure. Chem. Mater..

[6] Saito K., Inaguma K., Ogawa M., Ha P. T., Akiyama H., Yamaguchi S., Minokoshi H., Ogasawara M., Kato S. (2022). Lepidocrocite-type layered titanate nanoparticles as photocatalysts
for H_2_ production. ACS Appl. Nano
Mater..

[7] Sheng L., Liao T., Kou L., Sun Z. (2017). Single-crystalline
ultrathin 2D TiO_2_ nanosheets: a bridge towards superior
photovoltaic devices. Mater. Today Energy.

[8] Ma J., Reeves K. G., Porras
Gutierrez A.-G., Body M., Legein C., Kakinuma K., Borkiewicz O. J., Chapman K. W., Groult H., Salanne M. (2017). Layered lepidocrocite type structure isolated by revisiting
the sol–gel chemistry of anatase TiO_2_: a new anode
material for batteries. Chem. Mater..

[9] Hu Y., Li Y., Cheng J., Chen M.-s., Fu W., Liu B., Zhang M., Shen Z. (2020). Intercalation of carbon nanosheet
into layered TiO_2_ grain for highly interfacial lithium
storage. ACS Appl. Mater. Interfaces.

[10] Reeves K. G., Ma J., Fukunishi M., Salanne M., Komaba S., Dambournet D. (2018). Insights into
Li^+^, Na^+^, and K^+^ intercalation in
lepidocrocite-type layered TiO_2_ structures. ACS Appl. Energy Mater..

[11] Esmat M., Farghali A. A., El-Dek S. I., Khedr M. H., Yamauchi Y., Bando Y., Fukata N., Ide Y. (2019). Conversion of a 2D
lepidocrocite-type layered titanate into its 1D nanowire form with
enhancement of cation exchange and photocatalytic performance. Inorg. Chem..

[12] Elsanousi A., Elssfah E., Zhang J., Lin J., Song H., Tang C. (2007). Hydrothermal treatment duration effect
on the transformation of titanate
nanotubes into nanoribbons. J. Phys. Chem. C.

[13] Gao T., Fjellvåg H., Norby P. (2009). Crystal structures of titanate nanotubes:
a Raman scattering study. Inorg. Chem..

[14] Ma R., Bando Y., Sasaki T. (2003). Nanotubes
of lepidocrocite titanates. Chem. Phys. Lett..

[15] Dong Y., Wu Z.-S., Zheng S., Wang X., Qin J., Wang S., Shi X., Bao X. (2017). Ti_3_C_2_ MXene-derived sodium/potassium titanate
nanoribbons for high-performance
sodium/potassium ion batteries with enhanced capacities. ACS Nano.

[16] Badr H. O., El-Melegy T., Carey M., Natu V., Hassig M. Q., Johnson C., Qian Q., Li C. Y., Kushnir K., Colin-Ulloa E. (2022). Bottom-up, scalable synthesis of anatase nanofilament-based
two-dimensional titanium carbo-oxide flakes. Mater. Today.

[17] Badr H. O., Lagunas F., Autrey D. E., Cope J., Kono T., Torita T., Klie R. F., Hu Y.-J., Barsoum M. W. (2023). On the
Structure of One-Dimensional TiO_2_ Lepidocrocite. Matter.

[18] Wang L., Badr H. O., Yang Y., Cope J. H., Ma E., Ouyang J., Yuan L., Li Z., Liu Z., Barsoum M. W. (2023). Unique hierarchical
structures of one dimensional
lepidocrocite titanate with cation-exchangeable sites for extraordinary
selective actinide capture for water purification. Chem. Eng. J..

[19] Ouyang J., Badr H. O., Xiebin J., Yang Y., Wang L., Liu Y., Tu H., Shao D., Li Z., Yuan L. (2025). Selective
Th (IV) separation and immobilization by one-dimensional
lepidocrocite titanate. Chem. Eng. J..

[20] Mieles M., Walter A. D., Wu S., Zheng Y., Schwenk G. R., Barsoum M. W., Ji H. F. (2024). Hydronium-crosslinked
inorganic hydrogel
omprised of 1D lepidocrocite titanate nanofilaments. Adv. Mater..

[21] Panigrahi S., Badr H. O., Deuermeier J., Jana S., Fortunato E., Martins R., Barsoum M. W. (2024). Interfacial
engineering with one-dimensional
lepidocrocite TiO_2_-based nanofilaments for high-performance
Perovskite solar cells. ACS Omega.

[22] Wilson O. R., Carey M. S., Cope J. H., Badr H. O., Nantz J. M., ElMelegy T. A., Barsoum M. W., Magenau A. J. (2023). Repairable reinforced
composites of 1D TiO_2_ lepidocrocite mesoparticles and thiol-yne
click networks via alkylborane-initiated in situ polymerization. Cell Rep. Phys. Sci..

[23] Cardoza N. A., Badr H. O., Pereira R., Barsoum M. W., Kalra V. (2023). One-dimensional,
itania lepidocrocite-based nanofilaments and their polysulfide anchoring
apabilities in Lithium–Sulfur batteries. ACS Appl. Mater. Interfaces.

[24] Walter A. D., Schwenk G. R., Liu Y., Bugallo Ferron D., Wilk J. T., Ferrer L. M., Li C. Y., Hu Y.-J., Barsoum M. W. (2025). Concentration-dependent control of the band gap energy
of a low-dimensional lepidocrocite titanate. ACS Nano.

[25] Colin-Ulloa E., Martin J. L., Hanna R. J., Frasch M. H., Ramthun R. R., Badr H. O., Uzarski J. R., Barsoum M. W., Grimm R. L., Titova L. V. (2023). Electronic structure of 1D lepidocrocite TiO_2_ as revealed by optical absorption and photoelectron spectroscopy. J. Phys. Chem. C.

[26] Walter A. D., Schwenk G. R., Cope J., Lindsay A. J., Sudhakar K., Hassig M. Q., Ferrer L., Mininni A., Barsoum M. W. (2023). Adsorption
and self-sensitized photodegradation of rhodamine 6G and crystal violet
by one-dimensional titanium dioxide-based lepidocrocite under visible
light. Matter.

[27] Walter A. D., Benamor H., Ferrer L. M., Reji T., Curran T., Schwenk G. R., Hadji M., Creighton M. A., Barsoum M. W. (2024). Self-sensitized photodegradation
and adsorption of
aqueous malachite green dye using one-dimensional titanium oxide nanofilaments. iScience.

[28] Lagunas F., Bugallo D., Karimi F., Yang Y., Badr H. O., Cope J. H., Ferral E., Barsoum M. W., Hu Y.-J., Klie R. F. (2024). Ion-exchange effects
in one-dimensional lepidocrocite
TiO_2_: a cryogenic scanning transmission electron microscopy
and density functional theory study. Chem. Mater..

[49] Schwenk G. R., Walter A. D., Barsoum M. W. (2024). Solvent-driven
self-assembly of one-dimensional
lepidocrocite titanium-oxide-based nanofilaments. Nano Lett..

[50] Steiner T. (2002). The hydrogen
bond in the solid state. Angew. Chem., Int.
Ed..

[29] Zhang T., Yu S., Wu Y., Ibrahim M. A., Walter A. D., Schwenk G. R., Hu Y.-J., Barsoum M. W., Li C. Y. (2024). Tuning the 1D-to-2D
transition in lepidocrocite titanate nanofilaments via polymer wrapping. Matter.

[30] Boukhris S., Iacoban A. C., Ibrahim M., Badr H., Kuncser A. C., Neatu S., Neatu F., Barsoum M. W., Florea M., Constantin D. (2025). Structural analysis of colloidal titania-based ribbons
and their self-assembly upon drying. Small Struct..

[31] Ibrahim M. A., Walter A. D., Badr H. O., Schwenk G. R., Ibrahim A. M., Morris V. R., Boukhris S., Florea M., Constantin D., Barsoum M. W. (2025). Expanding the processing space of
quantum confined,
one-dimensional titania-based lepidocrocite nanofilaments. Matter.

[33] Sudhakar K., Karmakar A., Badr H. O., El-Melegy T., Hassig M. Q., Carey M., Masiuk S., Wu L., Qian Q., Kono T. (2023). One-dimensional, titania-based
lepidocrocite nanofilaments and their self-assembly. Matter.

[34] Kresse G., Furthmüller J. (1996). Efficient iterative schemes for ab initio total-energy
calculations using a plane-wave basis set. Phys.
Rev. B.

[35] Blöchl P. E. (1994). Projector
augmented-wave method. Phys. Rev. B.

[36] Perdew J. P., Burke K., Ernzerhof M. (1996). Generalized
gradient approximation
made simple. Phys. Rev. Lett..

[37] Mathew K., Sundararaman R., Letchworth-Weaver K., Arias T., Hennig R. G. (2014). Implicit
solvation model for density-functional study of nanocrystal surfaces
and reaction pathways. J. Chem. Phys..

[38] Momma K., Izumi F. (2011). VESTA 3 for three-dimensional visualization of crystal, volumetric
and morphology data. Appl. Crystallogr..

[39] Yang, Y. R. ; Lam, S. S. General AIMD congestion control. In Proceedings 2000 International Conference on Network Protocols; IEEE, 2000; pp 187–198.

[40] Guillemin F., Robert P., Zwart B. (2004). AIMD algorithms
and exponential functionals. Ann. Appl. Probab..

[41] Stukowski A. (2010). Visualization
and analysis of atomistic simulation data with OVITO–the Open
Visualization Tool. Modell. Simul. Mater. Sci.
Eng..

[42] Heyd J., Scuseria G. E., Ernzerhof M. (2003). Hybrid functionals
based on a screened
Coulomb potential. J. Chem. Phys..

[43] Wang V., Xu N., Liu J.-C., Tang G., Geng W.-T. (2021). VASPKIT: A user-friendly
interface facilitating high-throughput computing and analysis using
VASP code. Comput. Phys. Commun..

[44] Togo A. (2023). First-principles
phonon calculations with phonopy and phono3py. J. Phys. Soc. Jpn..

[45] Skelton J. M., Burton L. A., Jackson A. J., Oba F., Parker S. C., Walsh A. (2017). Lattice dynamics of the tin sulphides
SnS 2, SnS and Sn 2 S 3: vibrational
spectra and thermal transport. Phys. Chem. Chem.
Phys..

[46] Badr H. O., Natu V., Neaţu Ş., Neaţu F., Kuncser A., Rostas A. M., Racey M., Barsoum M. W., Florea M. (2023). Photo-stable, 1D-nanofilaments TiO_2_-based
lepidocrocite for photocatalytic hydrogen production in water-methanol
mixtures. Matter.

[47] Chemseddine A., Moritz T. (1999). Nanostructuring titania: control
over nanocrystal structure,
size, shape, and organization. Eur. J. Inorg.
Chem..

[48] Zhang J., Sun P., Jiang P., Guo Z., Liu W., Lu Q., Cao W. (2019). The formation mechanism
of TiO_2_ polymorphs under hydrothermal
conditions based on the structural evolution of [Ti (OH)_h_ (H_2_O)_6–h_]_4–h_ monomers. J. Mater. Chem. C.

[51] Pimentel, G. A. L. McClellan The hydrogen bond; WH Freemann and Company: San Francisco und London, 1960.

[52] Jeffrey, G. A. ; Jeffrey, G. A. An Introduction to Hydrogen Bonding; Oxford University Press: New York, 1997.

[53] Badr H. O., Cope J., Kono T., Torita T., Lagunas F., Castiel E., Klie R. F., Barsoum M. W. (2023). Self-assembly
of
1D lepidocrocite titania into 2D sheets and mesoporous particles. Matter.

[54] Ma R., Sasaki T. (2010). Nanosheets of oxides
and hydroxides: ultimate 2D charge-bearing
functional crystallites. Adv. Mater.:Compos.
Carbon, Pap. Symp..

[55] Lin C., Poncé S., Marzari N. (2022). General invariance and equilibrium
conditions for lattice dynamics in 1D, 2D, and 3D materials. npj Comput. Mater..

[56] Rems E., Anayee M., Fajardo E., Lord R. L., Bugallo D., Gogotsi Y., Hu Y. J. (2023). Computationally
guided synthesis
of MXenes by dry selective extraction. Adv.
Mater..

[57] Parker T., Zhang D., Bugallo D., Shevchuk K., Downes M., Valurouthu G., Inman A., Chacon B., Zhang T., Shuck C. E. (2024). Fourier-transform infrared
spectral library
of MXenes. Chem. Mater..

[58] Eriksson F., Fransson E., Erhart P. (2019). The Hiphive
Package for the extraction
of high-order force constants by machine learning. Adv. Theory Simul..

[59] Eivari H. A., Ghasemi S. A., Tahmasbi H., Rostami S., Faraji S., Rasoulkhani R., Goedecker S., Amsler M. (2017). Two-dimensional hexagonal
sheet of TiO_2_. Chem. Mater..

[60] Wang L., Wei D., Kang S., Xie X., Shi Y., Liu S. (2018). Two-dimensional
titania: Structures and properties predicted by first principle calculation. J. Phys. Chem. C.

[61] Sousa C., Illas F. (1994). Ionic-covalent transition in titanium
oxides. Phys. Rev. B.

