# Supplementary material for: Atomic Structure, Stability, Raman Modes, and Electronic Properties of Quantum-Confined One-Dimensional Lepidocrocite Titanate and Water: A First-Principles Study
Source: ACS Omega. 2026 Jan 30;11(6):9960–71. doi: 10.1021/acsomega.5c10764 (PMC12917683; doi:10.1021/acsomega.5c10764)
Supplement: Supplementary file 1 [file ao5c10764_si_001.pdf]

Supporting Information

Atomic structure, stability, Raman modes, and electronic properties of quantum-confined one-dimensional lepidocrocite titanate and water: A first-principles study

Yuanren Liu, David Bugallo[1], Michel W. Barsoum*, and Yong-Jie Hu*

Department of Materials Science and Engineering, Drexel University, Philadelphia, PA19104, USA

*corresponding authors: barsoumw@drexel.edu; yh593@drexel.edu.

[1] Current address: Decotek Automotive, Mullingar Rd, Collinstown, Co. Westmeath, N91 KHN1, Ireland

Atomic structures of unterminated 1DLs before and after relaxation

Unterminated 1DL structures with exposed (001) edges are inherently unstable due to dangling bonds, regardless of their width. Upon relaxation, the typical zig-zag TiO_6 octahedral framework of the lepidocrocite lattice either collapses or becomes highly distorted, as shown in Tables S1–S4.

Table S1: Unterminated 1C 1DL structures before, and after, DFT relaxation.

Structure before relaxation	Structure after relaxation
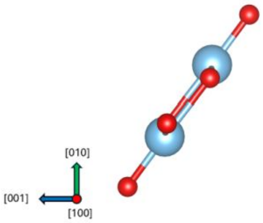	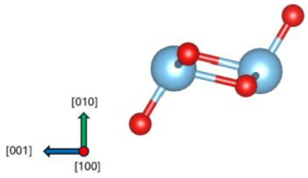
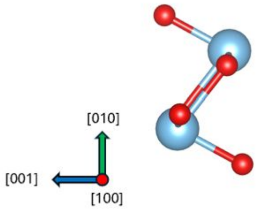	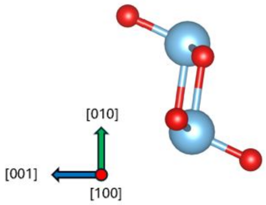
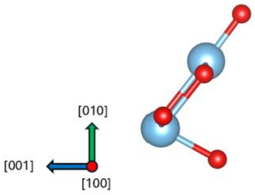	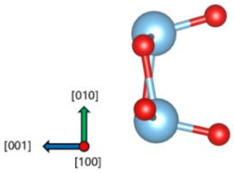
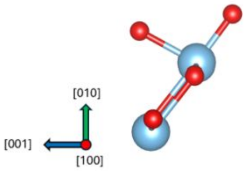	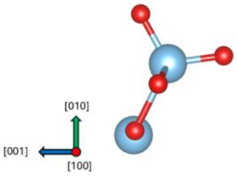

Table S2: Unterminated 2C 1DL structures before, and after, relaxation.

Structure before relaxation	Structure after relaxation
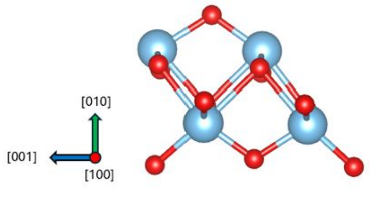	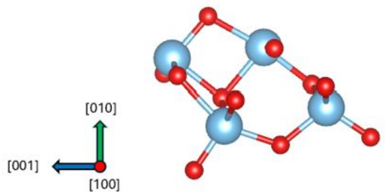
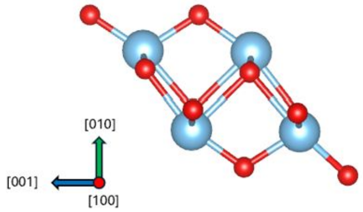	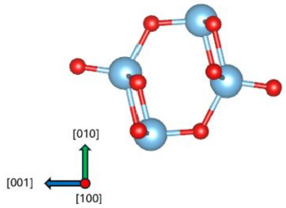
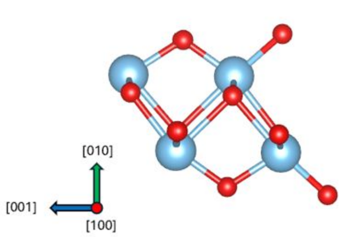	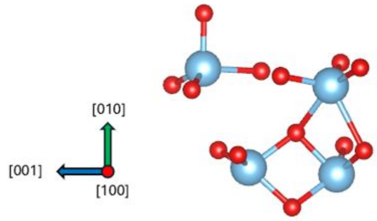
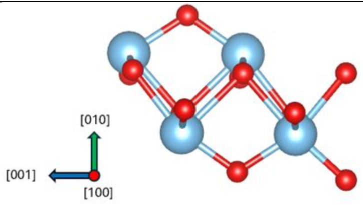	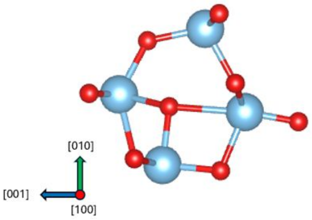

Table S3: Unterminated 3C 1DL structures before, and after, relaxation.

Structure before relaxation	Structure after relaxation
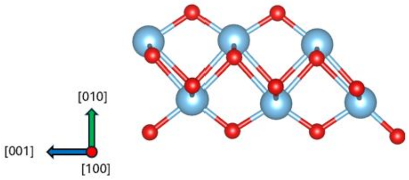	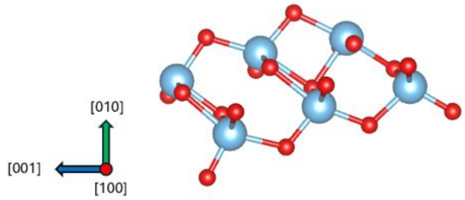
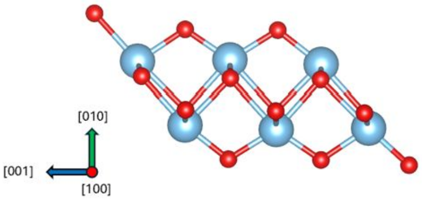	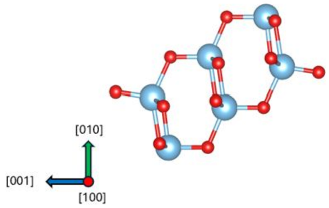

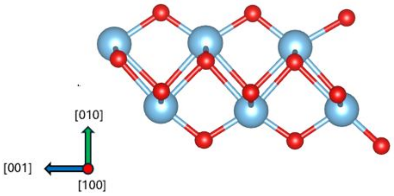	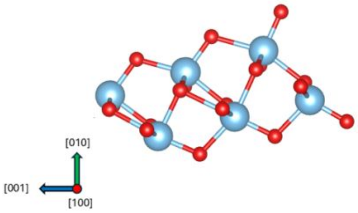
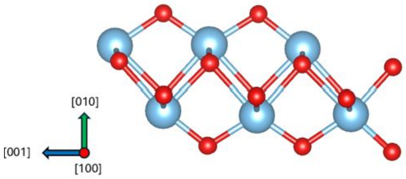	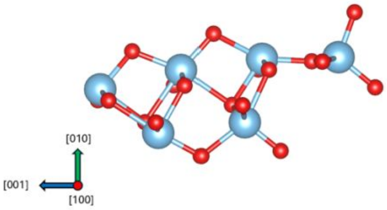

Table S4: Unterminated 4C 1DL structure before, and after, relaxation.

Structure before relaxation	Structure after relaxation
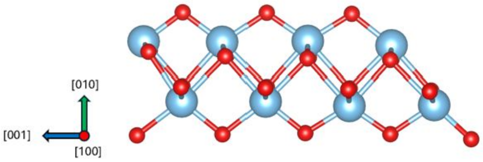	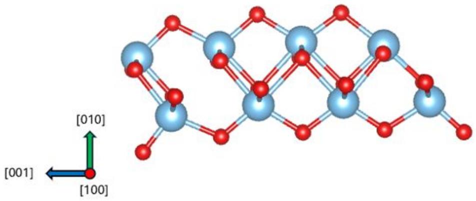
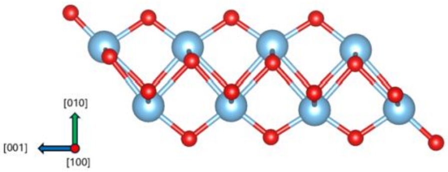	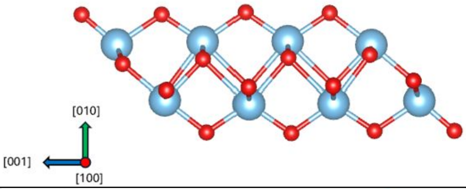
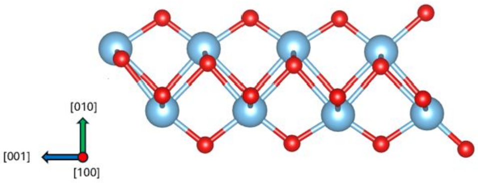	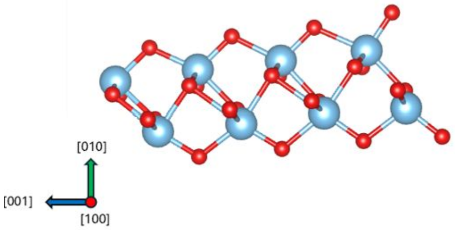
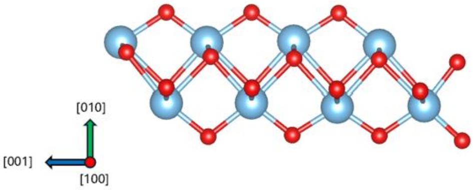	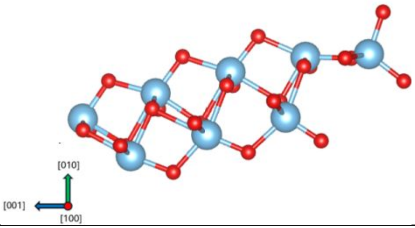

Terminated 1DL structures after relaxation

Tables S5-S8 summarize the various termination configurations for 1DLs, of which thermodynamic stability was evaluated through convex hull analysis in the present work.

Table S5: Terminated 1C 1DL structures before, and after, relaxation, along with their chemical formulae and formation energies, E_f, with reference to unterminated 1DL (column 4) and in reference to 2D lepidocrocite (column 5). Energy unit is eV/molecule.

Chemical formula ($m TiO_2 \cdot n H_2O$)	Structure before relaxation	Structure after relaxation	E_f relative to unterminated 1DL	E_f reference to 2D TiO_2
$2TiO_2 \cdot 2H_2O$	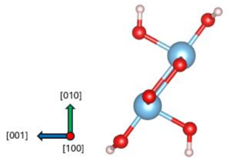	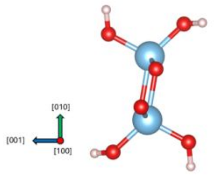	-0.3377	0.0870
$2TiO_2 \cdot 2H_2O$	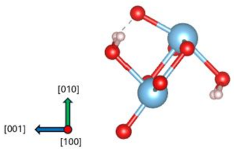	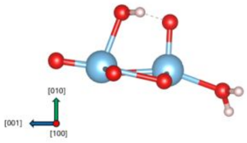	-0.1282	0.2965
$2TiO_2 \cdot 2H_2O$	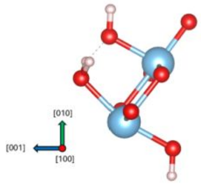	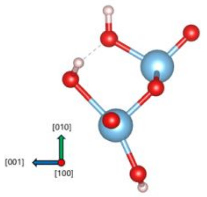	-0.2132	0.2115
$2TiO_2 \cdot 2H_2O$	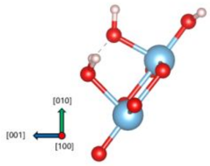	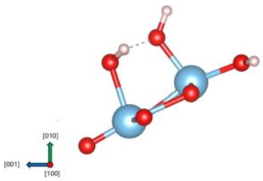	-0.1471	0.2775
$2TiO_2 \cdot 2H_2O$	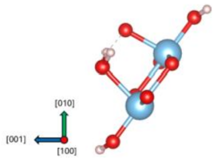	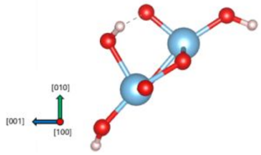	-0.1902	0.2375
$2TiO_2 \cdot 2H_2O$	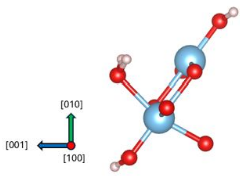	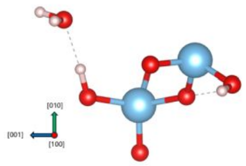	-0.0507	0.3740

$2TiO_2·3H_2O$	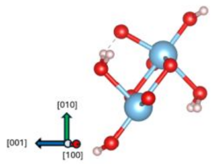	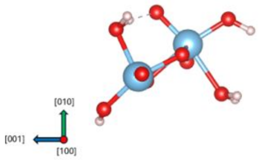	-0.2650	0.0748
$2TiO_2·3H_2O$	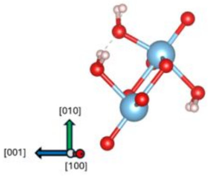	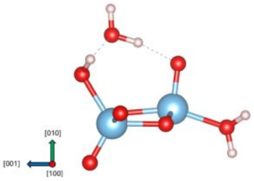	-0.1600	0.1798
$2TiO_2·4H_2O$	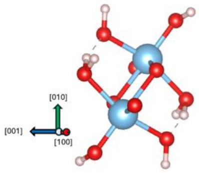	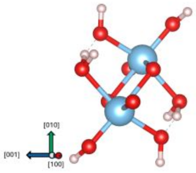	-0.3040	-0.0208
$2TiO_2·4H_2O$	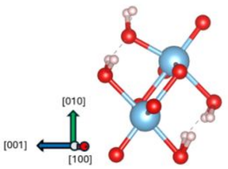	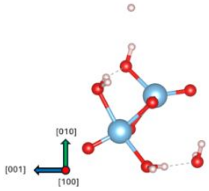	-0.0830	0.2001
$2TiO_2·4H_2O$	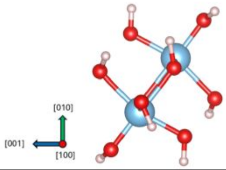	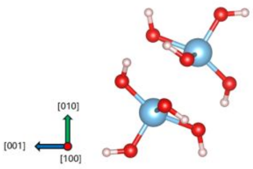	-0.0841	0.1990

Table S6: Terminated 2C 1DL structures before, and after, relaxation, along with their chemical formulae and formation energies, E_f, with reference to unterminated 1DL (column 4) and in reference to 2D lepidocrocite (last column). Energy unit is eV/molecule.

Chemical formula ($m\mathrm{TiO_2}\cdot n\mathrm{H_2O}$)	Structure before relaxation	Structure after relaxation	E_f reference to unterminated 1DL	E_f reference to 2D TiO_2
$4TiO_2\cdot 2H_2O$	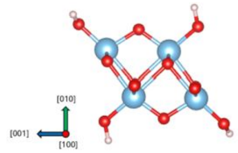	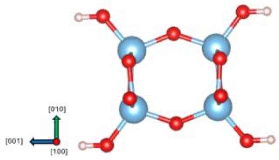	-0.5183	0.0388
$4TiO_2\cdot 2H_2O$	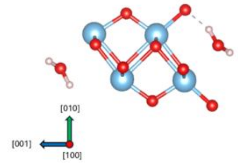	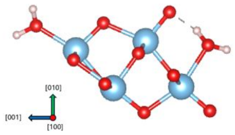	-0.2980	0.2591
$4TiO_2\cdot 2H_2O$	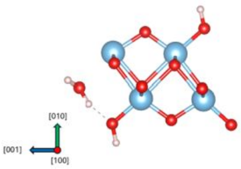	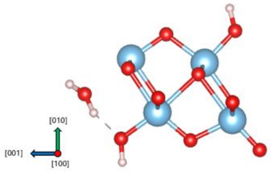	-0.3096	0.2475
$4TiO_2\cdot 2H_2O$	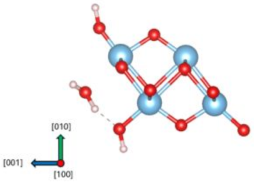	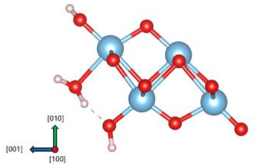	-0.3158	0.2413
$4TiO_2\cdot 2H_2O$	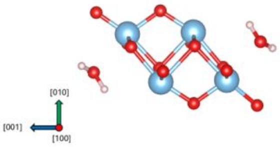	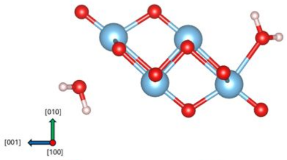	0.2253	0.7825
$4TiO_2\cdot 2H_2O$	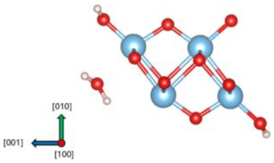	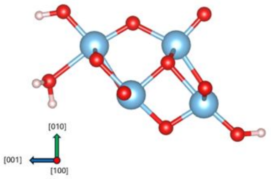	-0.3631	0.1940
$4TiO_2\cdot 3H_2O$	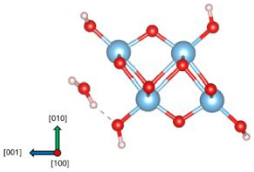	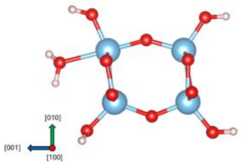	-0.4576	0.0100

4TiO$_2$·3H$_2$O	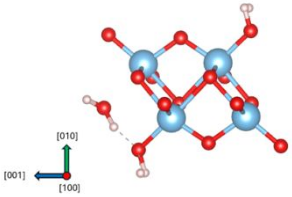	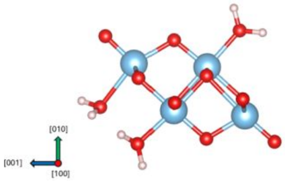	-0.2570	0.2205
4TiO$_2$·4H$_2$O	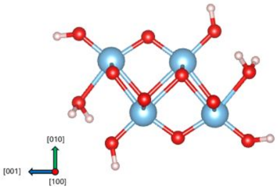	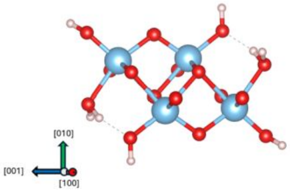	-0.4395	-0.0211
4TiO$_2$·4H$_2$O	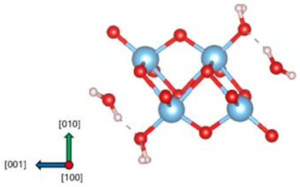	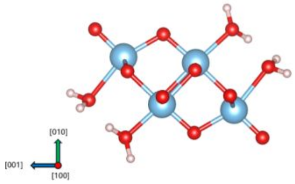	-0.2494	0.1685
4TiO$_2$·4H$_2$O	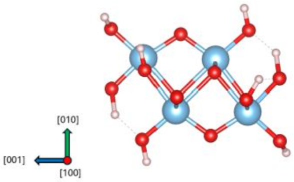	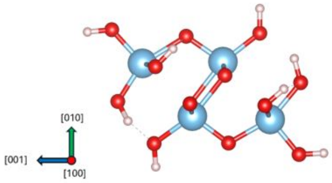	-0.2585	0.1594

Table S7: Terminated 3C 1DL structures before, and after, relaxation, along with their chemical formulae and formation energies, E$_f$, with reference to unterminated 1DL (column 4) and in reference to 2D lepidocrocite (last column). Energy unit is eV/molecule.

Chemical formula (mTiO$_2$·nH$_2$O)	Structure before relaxation	Structure after relaxation	E$_f$ reference to unterminated 1DL	E$_f$ reference to 2D TiO$_2$
6TiO$_2$·2H$_2$O	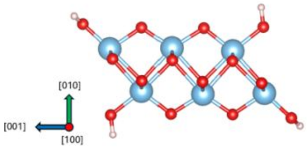	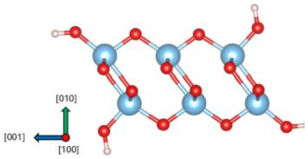	-0.3474	0.0414
6TiO$_2$·2H$_2$O	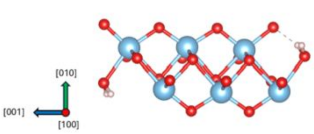	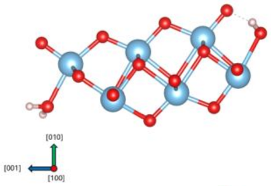	-0.1542	0.2345
6TiO$_2$·2H$_2$O	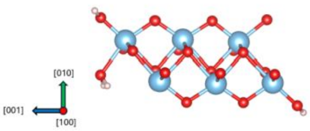	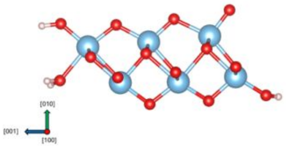	-0.2167	0.1720

$6TiO_2 \cdot 2H_2O$	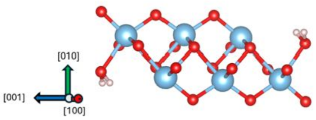	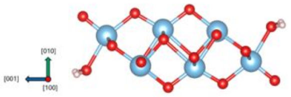	0.2372	0.6260
$6TiO_2 \cdot 2H_2O$	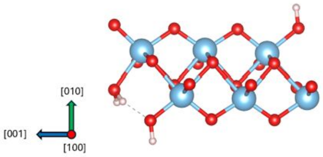	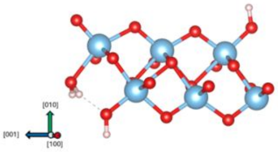	-0.2268	0.1618
$6TiO_2 \cdot 2H_2O$	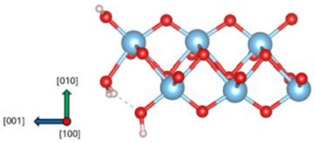	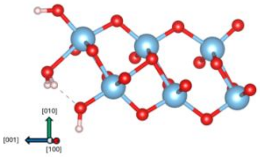	-0.1592	0.2295
$6TiO_2 \cdot 3H_2O$	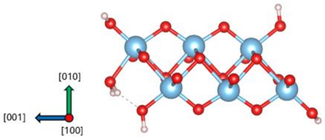	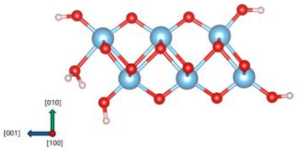	-0.3321	0.0064
$6TiO_2 \cdot 3H_2O$	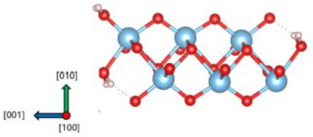	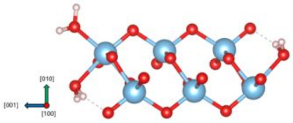	-0.2442	0.1013
$6TiO_2 \cdot 4H_2O$	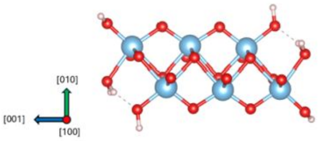	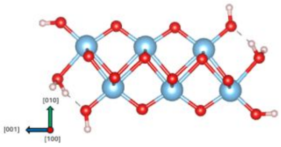	-0.3366	-0.0256
$6TiO_2 \cdot 4H_2O$	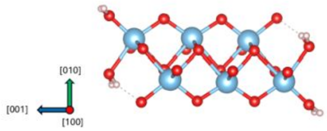	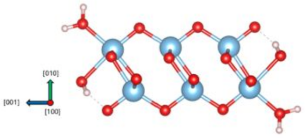	-0.2862	0.0248
$6TiO_2 \cdot 4H_2O$	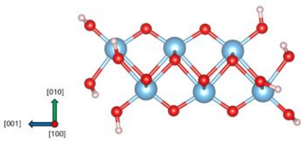	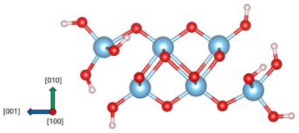	-0.1864	0.1246

Table S8: Terminated 4C 1DL structures before, and after, relaxation, along with their chemical formulae and formation energies, E_f, with reference to unterminated 1DL (column 4) and in reference to 2D lepidocrocite (last column). Energy unit is eV/molecule.

Chemical formula ($m TiO_2 \cdot n H_2O$)	Structure before relaxation	Structure after relaxation	E_f reference to unterminated 1DL	E_f reference to 2D TiO_2
$8TiO_2 \cdot 2H_2O$	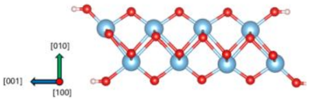	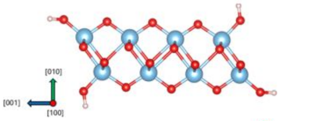	-0.2328	0.0485
$8TiO_2 \cdot 2H_2O$	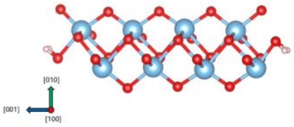	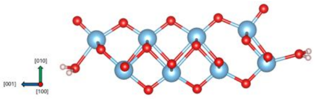	-0.0628	0.1944
$8TiO_2 \cdot 2H_2O$	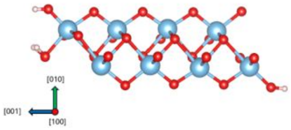	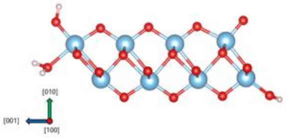	-0.1428	0.1144
$8TiO_2 \cdot 2H_2O$	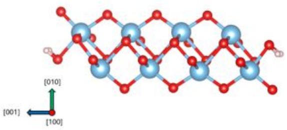	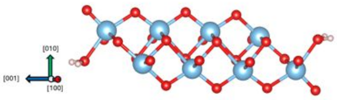	0.2372	0.4944
$8TiO_2 \cdot 2H_2O$	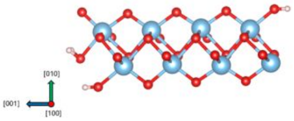	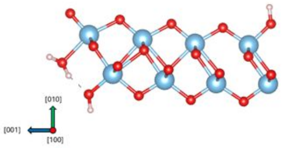	0.2072	0.4645
$8TiO_2 \cdot 2H_2O$	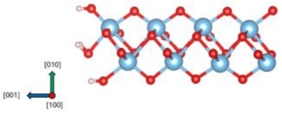	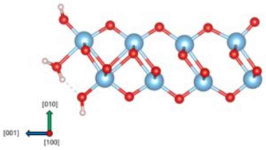	- 0.1728	0.0844
$8TiO_2 \cdot 3H_2O$	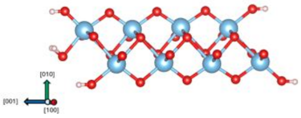	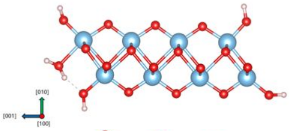	- 0.2256	0.0019
$8TiO_2 \cdot 3H_2O$	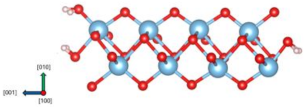	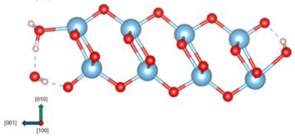	- 0.1629	0.0709
$8TiO_2 \cdot 4H_2O$	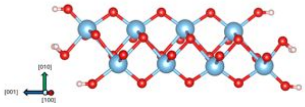	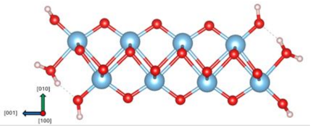	- 0.2380	- 0.0535
$8TiO_2 \cdot 4H_2O$	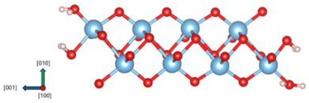	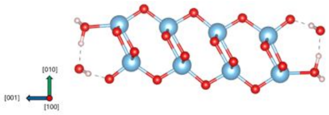	- 0.1522	0.0526

$8TiO_2{\cdot}4H_2O$	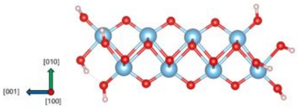	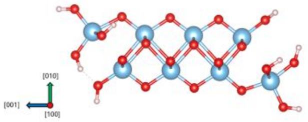	- 0.1132	0.1010

AIMD simulation of four H_2O terminated 1C 1DL structure at synthesis temperature (368.15K)

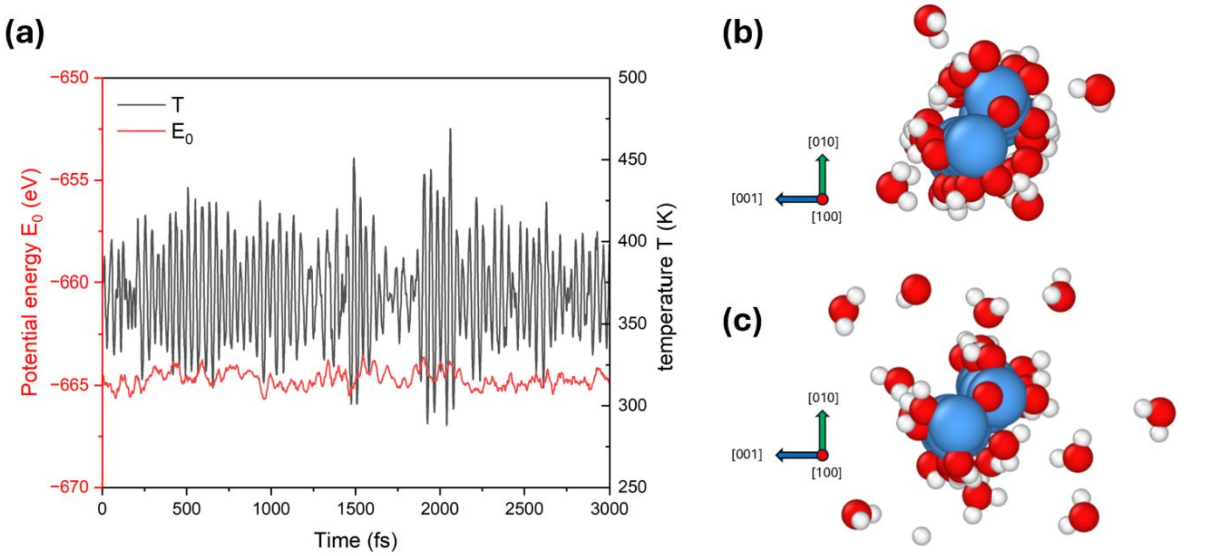

Figure S1: AIMD simulation of the thermodynamically stable, four H_2O terminated 1C 1DL structure at synthesis temperature (368.15K). (a): evolution of system potential energy and temperature as a function of simulation time for the 1C structure. (b): snapshots of the 1C atomic structures at the beginning of the simulation (0 fs). (c) same as (b), but at end of simulation (3,000 fs).

Electronic density of state (eDOS) and band structure dispersion of 2C, 3C, 4C 1DL structures

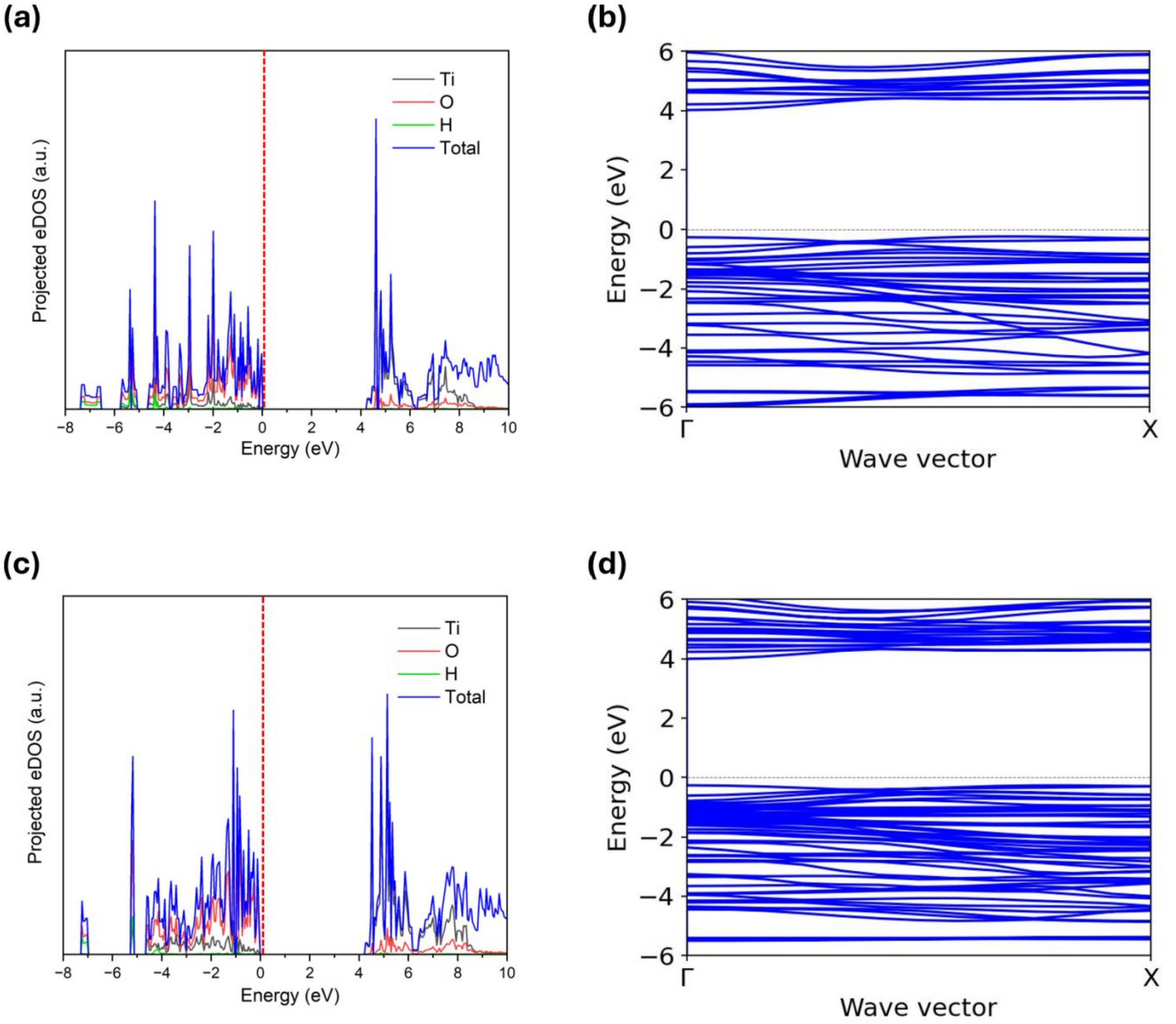

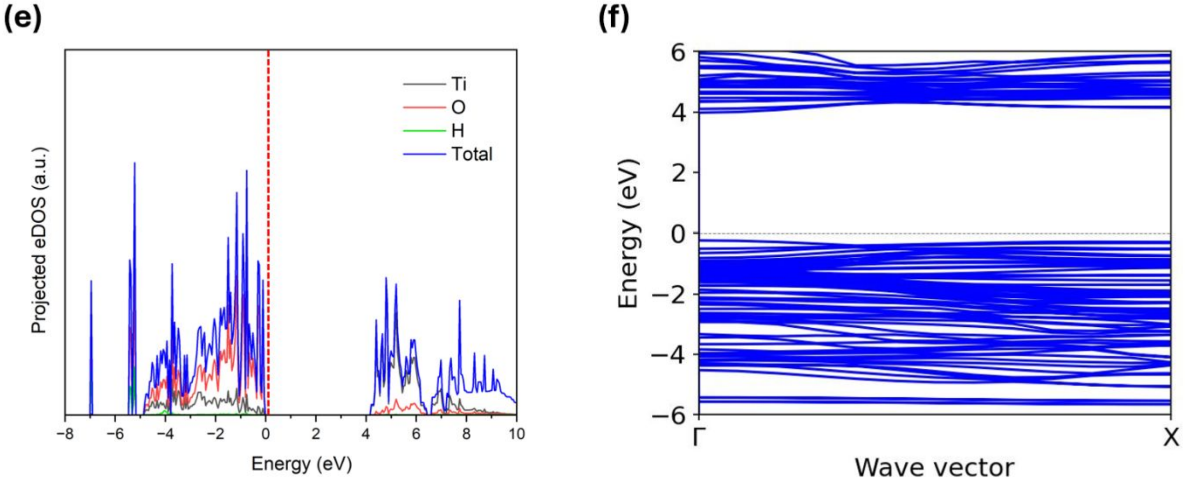

Figure S2: Total and projected electronic density of state (eDOS) of, (a) 2C, (c) 3C, and (e) 4C 1DL structures. Electron band dispersion of (b) 2C, (d) 3C, and (f) 4C 1DL structures.

Raman peak vibration modes of 2C, 3C 1DL structure and 2D lepidocrocite (2DL) TiO_2

Table S9: Vibration modes and peak positions of the theoretical Raman spectra of the 2C and 3C 1DLs and 2D lepidocrocite TiO_2.

Peak Position (cm^{-1})	Structure	Vibration origins	Vibration modes
789	2C	Backbone (Ti-O)	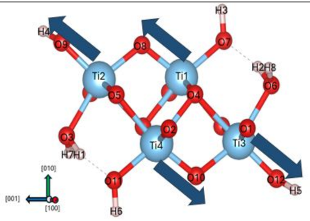
783	3C	Backbone (Ti-O)	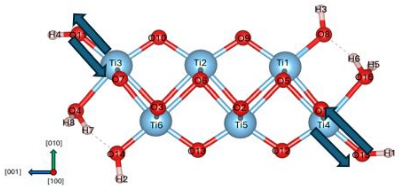
755	2C	Backbone (Ti-O)	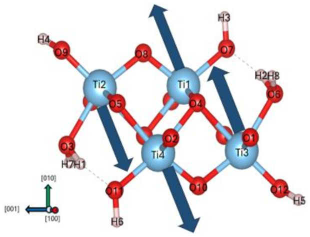
711	2D TiO_2	Backbone (Ti-O)	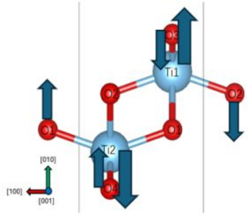
667	2C	Backbone (Ti-O)	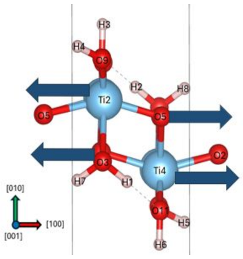

667	3C	Backbone (Ti-O)	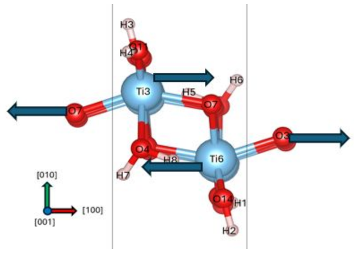
665	2DL	Backbone (Ti-O)	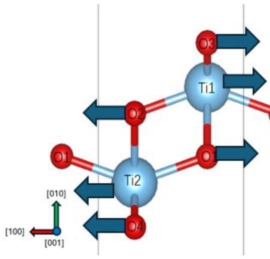
622	3C	Termination (-H, -OH, or H_2O)	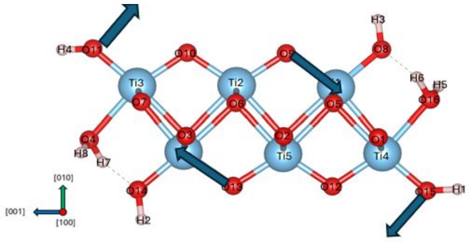
599	2C	Backbone (Ti-O)	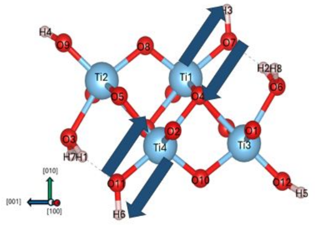
579	3C	Backbone (Ti-O)	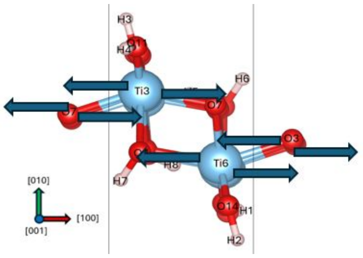
574	2C	Backbone (Ti-O)	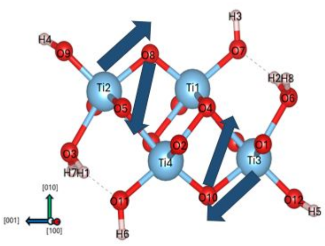

573	3C	Backbone (Ti-O)	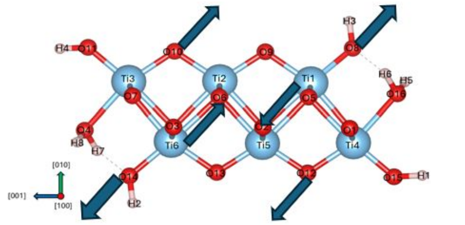
570	2DL	Backbone (Ti-O)	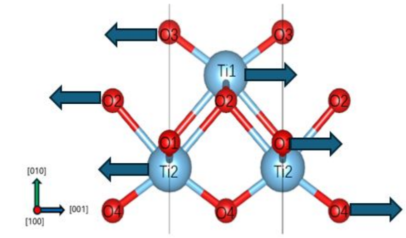
513	2C	Termination (-H, -OH, or H_2O)	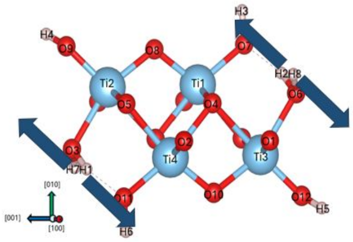
500	2C	Termination (-H, -OH, or H_2O)	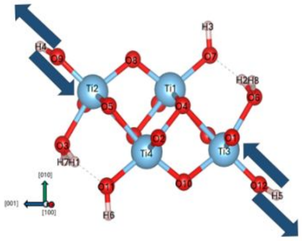
491	3C	Termination (-H, -OH, or H_2O)	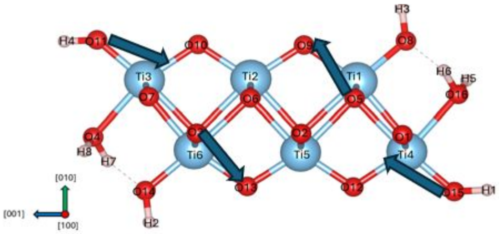
470	2C	Termination (-H, -OH, or H_2O)	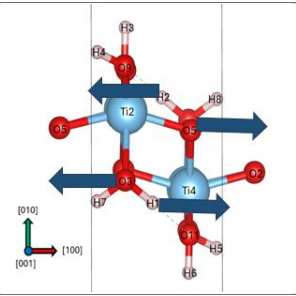

463	3C	Termination (-H, -OH, or H_2O)	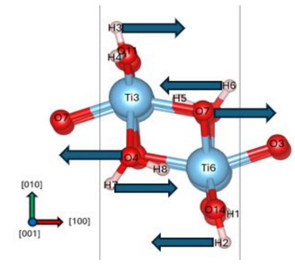
450	2DL	Backbone (Ti-O)	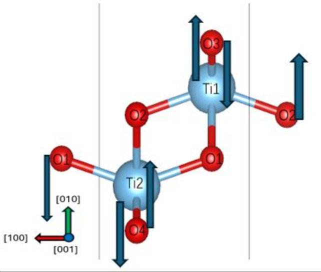
400	2C	Termination (-H, -OH, or H_2O)	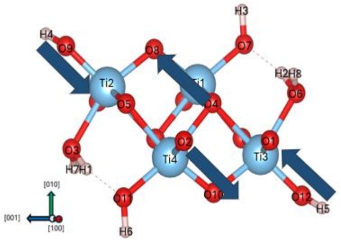
380	3C	Termination (-H, -OH, or H_2O)	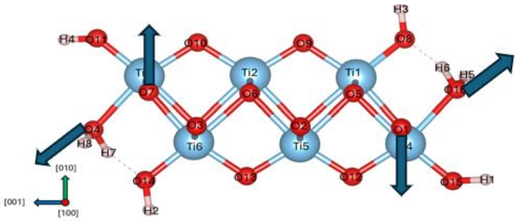
353	2C	Termination (-H, -OH, or H_2O)	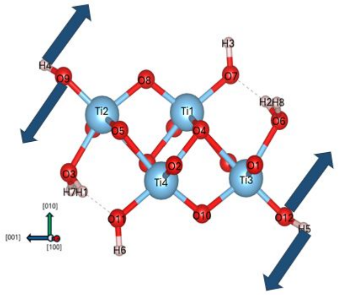
327	2C	Backbone (Ti-O)	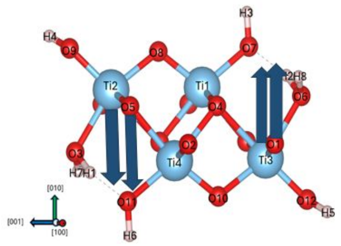

288	2C	Backbone (Ti-O)	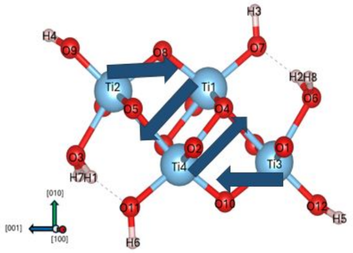
286	2DL	Backbone (Ti-O)	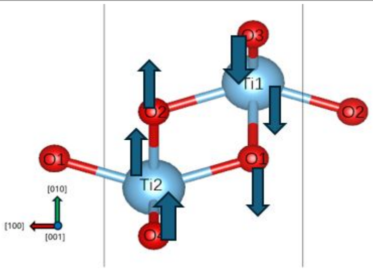
279	3C	Backbone (Ti-O)	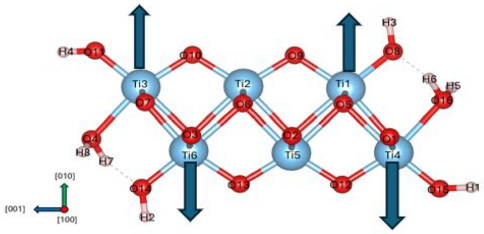
267	2C	Backbone (Ti-O)	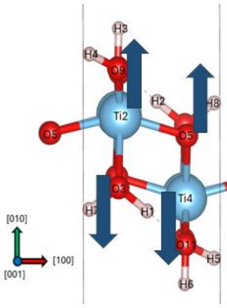
236	2C	Backbone (Ti-O)	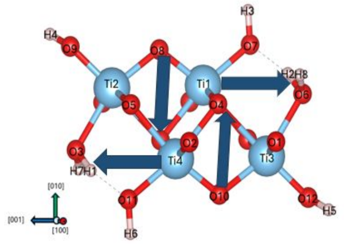
227	2C	Backbone (Ti-O)	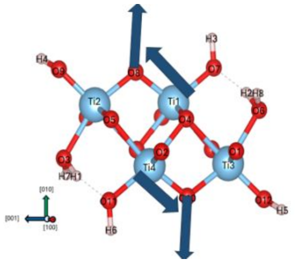

217	3C	Backbone (Ti-O)	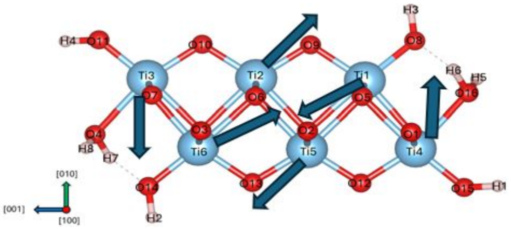
215	2DL	Backbone (Ti-O)	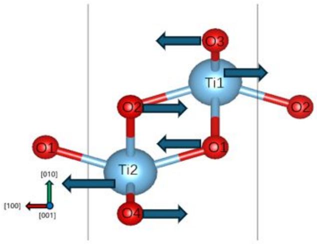
201	2C	Backbone (Ti-O)	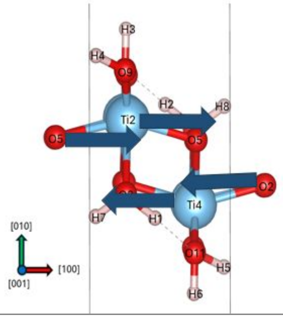
194	3C	Backbone (Ti-O)	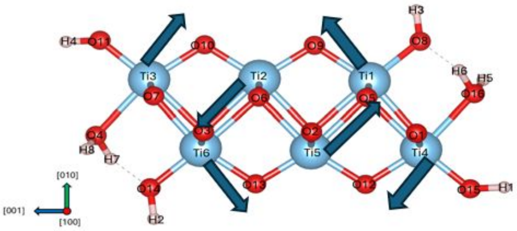
189	2C	Backbone (Ti-O)	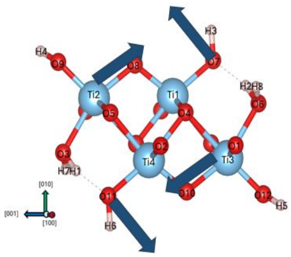
146	2C	Backbone (Ti-O)	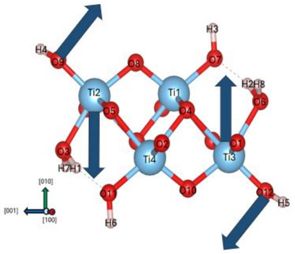

143	3C	Backbone (Ti-O)	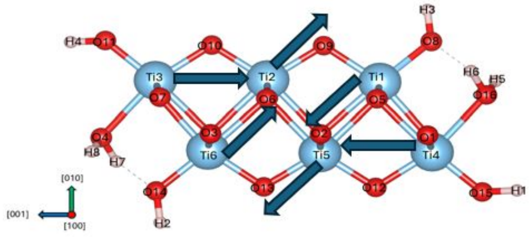
95	2C	Backbone (Ti-O)	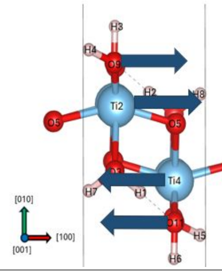
73	3C	Backbone (Ti-O)	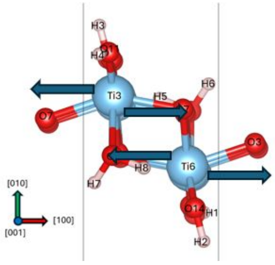
7	3C	Backbone (Ti-O)	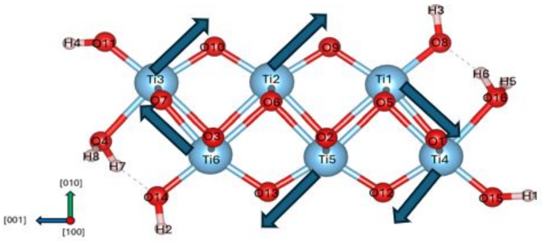
